# Measuring individual differences in the speed of attention using the distractor intrusion task

**DOI:** 10.3758/s13428-025-02916-8

**Published:** 2026-01-23

**Authors:** Alon Zivony, Claudia C. von Bastian, Rachel Pye

**Affiliations:** 1 School of Psychology, University of Sheffield, Sheffield, UK; 2 School of Psychology and Clinical Language Sciences, University of Reading, Reading, UK

**Keywords:** Individual differences, Distractor intrusions, Visual search, RSVP, Attentional blink

## Abstract

How quickly we attend to objects plays an important role in navigating the world, especially in dynamic and rapidly changing environments. Measuring individual differences in attention speed is therefore an important, yet challenging, task. Although reaction times in visual search tasks have often been used as an intuitive proxy of such individual differences, these measures are limited by inconsistent levels of reliability and contamination by non-attentional factors. This study introduces the rate of post-target distractor intrusions (DI) in the rapid serial visual presentation (RSVP) paradigm as an alternative method of studying individual differences in the speed of attention. In RSVP, a target is presented for a brief duration and embedded among multiple distractors. DIs are reports of a subsequent distractor rather than the target and have previously been shown to be associated with the speed of attention. The present study explored the reliability and validity of DI rates as a measure of individual differences. In three studies, DI rates showed high internal consistency and test–retest reliability over a year (>.90), even with a short task administration of only about 5 minutes. Moreover, DI rates were associated with measures related to attention speed, but not with unrelated measures of attentional control, reading speed, and attentional blink effects. Taken together, DI rates can serve as a useful tool for research into individual differences in the speed of attention. Links to a downloadable and easily executable DI experiment, as well as a brief discussion of methodological considerations, are provided to facilitate such future research.

## Introduction

How quickly people notice important events in dynamic environments plays a critical role in guiding our actions and determining their outcomes. For example, as any driver knows, a tiny delay in detecting a sudden change on the road can mean the difference between safely stopping the car and a fatal accident. Visual selective attention is crucial for rapid perception of such hazards, as it enables prioritized processing of potentially important events in the changing environment. Accordingly, much effort has been devoted to studying the various factors that affect how quickly attention is deployed (see, e.g., Wagner et al., 2024; Wolfe, 2020, for reviews). In the current study, we focus on one such factor: individual differences.

The study of individual differences in cognition has the potential to bridge insights from well-controlled lab experiments with predictions of behavior in the real world. For example, individual differences in the speed of attention can advance understanding as to why some people are slower to detect road hazards or are more prone to have car accidents (e.g., Barragan & Lee, 2021). However, despite their importance, individual differences in the speed of attention are still poorly understood.

Selective attention research has overwhelmingly relied on an experimental approach that tends to emphasize universal regularities in attention mechanisms, rather than variability between individuals. Ideally, progress in one research tradition, experimental or individual differences, would translate to progress in the other. However, various challenges limit this kind of cross-fertilization (Cronbach, 1957). One recently discussed challenge is that measures suitable for experimental research may not be suitable for individual differences research, due to their restricted reliability (Hedge et al., 2018a; Rouder & Haaf, 2019).

Take, for example, the Visual Search task, arguably the most popular task used to study the determinants of the speed of attention (Nakayama & Martini, 2011; Wolfe, 2020). In Visual Search, participants are presented with a static display where a target is surrounded by distractors. Performance is most often measured using participants’ reaction times (RTs). Linking between RTs and the speed of attention seems straightforward: participants who are slower to detect and attend to the target should take longer to react to it. However, RTs reflect the endpoint of multiple processes, not just attention. Therefore, when a difference in RTs is observed between conditions or between individuals, it is often unclear which of the processes is responsible for this difference (Palmer et al., 2011). To isolate attention-related differences from other processes, attention researchers often use sophisticated experimental designs where two or more conditions differ in one key aspect, and then calculate measures derived from the difference between scores on these conditions. These kinds of difference measures are useful because they control for any differences that are unaffected by the manipulation, such as response-selection mechanisms and general individual differences in processing speed. For example, changing the number of distractors in a Visual Search display allows one to calculate the average speed of discarding a distractor (a search slope; Wolfe, 2001). If a participant is generally slower to respond, this irrelevant source of variability should emerge in all measurements and therefore should be cancelled out when the slope is calculated. Thus, calculating difference scores is key for the experimental study of the speed of attention. At the same time, the difference score method can result in poor reliability, as it relies on subtracting strongly correlated measures (participants’ raw RTs) from each other (Caruso, 2004; Cronbach & Furby, 1970). Indeed, while very few papers report the reliability of search slopes (Wagner et al., 2024), the ones that do often report restrictively low values (e.g., Sisk et al., 2022).

Research on individual differences in the speed of attention is impeded by these issues. Intuitively, it seems reasonable that some participants deploy their attention more quickly than others, and that RTs can be used to measure these differences. In practice, individual differences in raw RT scores are confounded by additional intervening processes and are also prone to speed–accuracy trade-offs (Draheim et al., 2019). Difference scores, which are meant to resolve some of these issues, are highly prone to reliability issues (Hedge et al., 2018a). Together, these issues limit the usability and interpretability of RTs in research into individual differences in the speed of attention. The purpose of this study is to overcome these challenges. We do so by introducing a new measure for individual differences in the speed of selective attention: distractor intrusion rates.

### Distractor intrusions as a measure of the speed of attention

Other than the Visual Search task, the speed of attention has been studied using the rapid serial visual presentation (RSVP) paradigm. In RSVP, participants are asked to identify one or more targets among multiple objects that appear and disappear in rapid succession (usually around 100 ms per object, i.e., 10 Hz) at the same location. Thus, whereas Visual Search requires the (rapid) deployment of attention to the right location in space, performance in the RSVP paradigm depends on rapidly allocating attention to the right object at the right moment in time. Importantly, responses in the RSVP paradigm are usually given without time pressure. Hence, unlike Visual Search (and other RT-based experiments), performance in RSVP is largely assumed to be impervious to variability in the speed of response selection mechanisms or to individual differences in overall response speed.

**Fig. 1:**
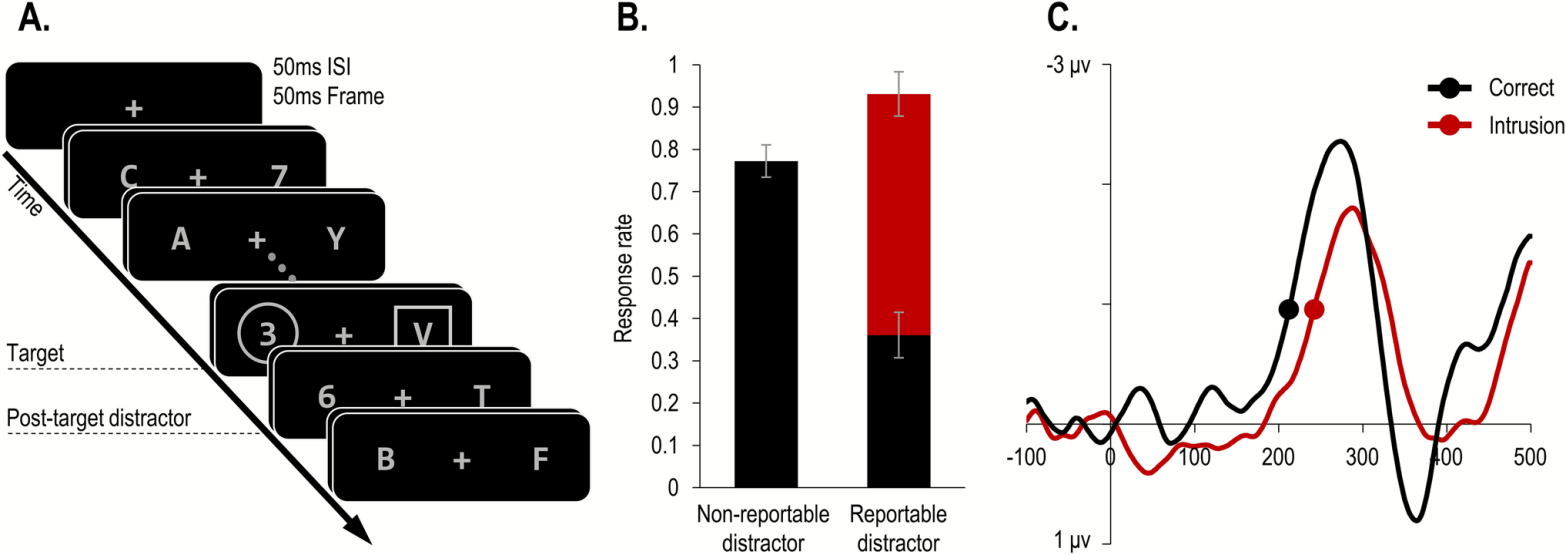
Illustration of the stimulus sequence in the tasks analyzed in Study 1 (**A**), including typical behavioral results (**B**) and electrophysiological (**C**) results. **A** Participants had to report a target from a pre-specified alphanumeric category (digit or letter) in one of two RSVP streams, defined by a predefined selection feature (circle or square). In the current example, the target is the digit inside the circle cue. At the same location as the target, the frame contained a category-matching post-target distractor (e.g., digit), which therefore allowed for distractor intrusion responses. **B** Behavioral results from Zivony and Eimer (2021). When the target is followed by a nonreportable distractor, accuracy (*black bar*) is high. In contrast, when the target is followed by a reportable distractor, accuracy is substantially lowered, and distractor intrusions are common. **C** N2pc results from Zivony and Eimer (2021). When the target is followed by a reportable distractor, the onset of the N2pc is slowed on trials where participants make intrusion errors relative to correct responses. Reprinted with permission

Since its inception, performance in the RSVP paradigm has been associated with the speed of attention (Lawrence, 1971; Broadbent & Broadbent, 1987). Broadbent and Broadbent (1987) suggested that performance in the RSVP paradigm relies on two processes: detection and identification. When a unique and salient target is presented in the RSVP paradigm, detection occurs rapidly and with little variability. In contrast, the attentional selection process responsible for identification and encoding is temporally variable and, therefore, can sometimes be delayed. This delay leads to the perception and report of the wrong object. For example, if the target is defined as a digit inside a disk (Fig. 1A), participants will often report seeing the immediately following (post-target) distractor digit (Fig. 1B). Such distractor reports have been documented in numerous studies using various types of stimuli (e.g., Adler & Intraub, 2021; Botella & Eriksen, 1992; Botella et al., 2001; Vul et al., 2008; Zivony & Eimer, 2020, 2024a, 2024b).

Moreover, studies have documented that distractor reports do not emerge merely due to guessing or response bias. Rather, these responses reflect occasions where the distractor’s identity is encoded to working memory instead of (or alongside) the target’s identity (Recht et al., 2019; Vul et al., 2009; Zivony & Eimer, 2020). Distractor reports in RSVP tasks reflect a genuine temporal binding error between the feature that defines the target (e.g., the selection cue) and the distractor (Zivony & Eimer, 2024a). Accordingly, these responses have been aptly labelled “distractor intrusions” (Botella & Eriksen, 1992), as they reflect an involuntary intrusion of the distractor information into conscious perception.

Over the last three decades, many studies have used distractor intrusion (hereafter *DI*) rates (and measures derived from DI responses) as an index of the speed of attention (e.g., Chun, 1997; Vul et al., 2008; Goodbourn et al., 2016; Ludowici & Holcombe, 2021). In these studies, a higher rate of post-target DIs was assumed to reflect occasions where attention was delayed. However, direct evidence to support this association has only recently been obtained. First, DI rates have been shown to be affected by manipulations known to affect the speed of attention. For example, target attentional selection is faster when there is more certainty about the target’s spatial location (e.g., Foster et al., 2020) and temporal position (e.g., MacKay & Juola, 2007), and when the target-defining feature is easier to detect (e.g., Wolfe & Horowitz, 2017). Correspondingly, DI rates increase when the target can appear in more locations (i.e., more RSVP streams), when the target’s temporal position is less predictable, and when the selection cue is less salient (Ludowici & Holcombe, 2021; Zivony & Eimer, 2021; 2023). Second, DIs have been associated with a delay to the N2pc, a well-known electrophysiological marker of selective attention (Eimer, 1996; Woodman & Luck, 1999). The onset of the N2pc has been closely linked with the timing of selective attention (Callahan-Flintoft et al., 2018; Zivony et al., 2018). In line with the view that DIs are more likely to occur when attention is slowed (e.g., Chun, 1997), several studies reported that these responses are consistently associated with a delay to the N2pc’s onset, relative to correct responses (Zivony & Eimer, 2020, 2021; see Fig. 1C).

### A framework for conceptualizing attention

The studies that report an association between DIs and the speed of attention fit well with Broadbent and Broadbent’s (1987) account of this phenomenon (unlike other theoretical accounts, see Zivony & Eimer, 2020 for a detailed discussion). However, one issue with Broadbent and Broadbent’s account is its reliance on the problematic concept of “attentional selection”. It has been argued by many that attentional selection (and attention more broadly) is a flawed and vague concept that can result in circular logic (Anderson, 2011; Di Lollo, 2018; Rosenholtz, 2024). Moreover, the conceptualization of attentional selection in Broadbent and Broadbent’s (1987) account (as in many other accounts) necessitates the adoption of implausible assumptions, like the notion that attention is a temporally discrete process (Zivony & Eimer, 2022). Given these issues, it is unsurprising that some have expressed skepticism about the merit of relying on the concept of attentional selection in scientific investigation (Anderson, 2011; Di Lollo, 2018; Rosenholtz, 2024).

We suggest that a coherent framework for (the speed of) attention is possible if one rejects the view that equates attention to selection, in favor of a view of attention as modulation (Fazekas & Nanay, 2021). Elsewhere, we provided a detailed discussion of why we believe this view resolves the theoretical problems with the concept of attention (Zivony & Eimer, 2022). What follows here is a brief description of our view, aimed at clarifying the concepts used in this work.

According to the attention-as-modulation view, perception occurs gradually and can be viewed as an evidence accumulation process. Encoding is the process of stabilizing a fragile sensory representation, making it resilient to competition from other sensory signals; this occurs only when sufficient sensory evidence about an object is accumulated. Attention is a family of modulatory processes that unfold over time (a “diachronic” process) and continuously modulate the efficiency of perceptual processing, thereby increasing the likelihood of encoding. Attention is associated with selectivity for two reasons. First, attention is deployed selectively: when and where attention is deployed is based on prior computations of salience and a stimulus’ match to the observer’s goals (Luck et al., 2021). Second, attention is not merely dependent on selective processes but also results in further selectivity. Specifically, attention biases the perceptual competition between multiple sensory signals, thereby increasing the chance that some will be encoded and others will not (Wyble et al., 2011). However, attention and selection are not one and the same: once deployed, attention modulates sensory signals indiscriminately.

This framework allows for a coherent association between the speed of selective attention and DIs. In RSVP tasks, most distractor stimuli are not encoded because their fragile sensory representation is overridden by preceding and following items. Detection of the target (i.e., sufficient evidence is accumulated about the presence of its defining feature) results in “attentional engagement”, a ballistic and transient attentional modulation. Attentional engagement substantially amplifies the processing of all stimuli at the target’s location for a short amount of time, which greatly enhances the likelihood that these stimuli will be encoded (whether they are relevant to the task or not). While attention is not a unitary process, throughout this work, we use the term “attention” and “the speed of attention”, to refer specifically to this transient modulation that follows the target’s detection. It is the timing of this attentional process, which is indexed by the N2pc component (Callahan-Flintoft et al., 2018; Zivony et al., 2018), that determines whether the target or distractor will be reported in the DI task. Correct responses occur when attentional engagement is triggered quickly (indexed by an early N2pc), which allows the target’s processing to be enhanced before its sensory trace is overridden by the following item. In contrast, DI responses occur when attentional engagement is slow (indexed by a later N2pc), which means that the post-target item benefits from more indiscriminate amplification than the target.

Finally, this framework suggests that temporal selectivity is the outcome of multiple interrelated yet distinct processes. Dissociating attention from these processes is a challenging task, but not an impossible one. For example, while attention and encoding are closely related, they are not one and the same. Attention modulates perceptual processing which, in turn, promotes encoding. However, various factors (including individual differences) have been found to affect the speed at which an object is encoded, independently of attentional modulations (Martens et al., 2006; Zivony & Eimer, 2024b). As will become apparent later, this is an important feature of our framework, as it will allow for pinpointing which processes are reflected by individual differences in DI rates.

### The current study

Previous research has shown that both experimental manipulations and trial-by-trial variability can affect the speed of attention, and consequently, affect DI rates. However, to date, these studies did not consider the possibility of consistent individual differences as a factor that affects the speed of attention. This is not surprising given that, like most of the field of visual attention, research into DIs has generally relied on an experimental approach. Therefore, it is still unclear whether people consistently vary in their speed of attention and whether DI rates could be a suitable measure for individual differences research.

In the current study, we aimed to provide a thorough test of DIs in a standardized task as a measure of individual differences in the speed of attention. Our first goal was to test the reliability of the DI rate measure, i.e., the likelihood of reporting a post-target distractor instead of the target. A strong test of a measure’s reliability requires a demonstration of both within-session reliability (i.e., internal consistency) as well as between-session (i.e., test–retest) reliability. In Study 1, we examined within-session reliability in previously collected data, and ran a simulation based on these data to determine the minimum number of trials and participants required for achieving high within-session reliability. In Studies 2 and 3, we collected new data which allowed us to examine within-session reliability as well as test–retest reliability in two sessions, 1 week apart and 1 year apart.

Our second goal was to provide additional tests examining the validity of DI rates as a measure of individual differences in the speed of attention. First, we examined whether DI rates are associated with other constructs that are not directly related to the speed of attention. Specifically, we tested whether individual differences in DI rates were associated with the attentional blink (Study 2A), reading abilities (Study 2B), and attentional control (Study 3A). Strong correlations between these measures and DI rates would indicate that these measures index similar processes – and, therefore, that DIs do not reflect a distinct cognitive process. In contrast, weak or no correlations between DI rates and these measures would suggest that DI rates tap into the speed of attention as a separate process. Second, we examined whether DI rates predict performance on measures that are directly related to the speed of attention: overall RTs in standard attention tasks (Study 3A) and errors in a time judgment task (Study 3B). Here, a correlation would suggest that DI rates assess the same cognitive process as other measures assessing the speed of attention.

To preview our results, we found DI rates to be highly reliable (≥.90), both within and between sessions, even with small sample sizes and relatively few trials. We also found that DI rates correlate with overall RT in standard attention tasks and with Time Judgment performance, but not with the other measures employed in this study. We argue that these results allow us to link DI rates and the speed of attention, rather than similar constructs (e.g., the speed of encoding). These findings open the door to new and potentially fruitful research using DI rates to examine the relationship between the speed of attention, other psychological variables, and real-world behavior.

## Study 1

In the first study, we examined the number of trials and participants required to achieve adequate levels of within-session reliability in a DI task. To do so, we combined data from previously conducted experiments and followed the down-sampling method developed by Xu et al. (2018) to measure the average within-session reliability for various combinations of trial and participant numbers. Both factors can substantially affect a measure’s reliability, which in turn can affect the ability to observe correlations between measures and reach valid conclusions about the speed of attention. Determining the minimum number of trials and participants is also important from a practical and methodological perspective. Unnecessarily long studies waste participants’ time and researchers’ funds. Moreover, shorter studies reduce participant fatigue and increase participant concentration, which is an important methodological consideration when employing large batteries of tasks and when studying special populations.

### Method

The current study was a reanalysis of datasets collected in three previous studies (Zivony & Eimer, 2020, Experiments 1–4; 2021, Experiments 1–2; 2023, Study 1), and the paradigm is fully described in these papers. The following is a description only of the information relevant to the particular reanalysis conducted here.

#### Participants

A total of 119 participants (73 women, 46 men, 0 non-binary) whose age ranged from 18 to 57 (*M*_age_ = 26.75 years, *SD*_age_ = 8.09) were included in the sample. All participants had normal or corrected-to-normal vision.

#### Apparatus

Stimuli were presented on a 24-in BenQ monitor (100 Hz; 1920 x 1080 screen resolution) attached to a SilverStone PC, with participant viewing distance at approximately 80 cm. Manual responses were registered via a standard computer keyboard.

#### Procedure

Participants had to report as accurately as possible the identity of an alphanumeric character that appeared inside a prespecified shape (circle or square; selection feature). For most participants (*n* = 103) the target was always a digit, whereas, for the rest (*n* = 16), the target was a letter. These targets were presented in one of two RSVP streams (on the left and right side of fixation). A critical feature of the trials analyzed here is that the distractor that appeared immediately following the target (post-target distractor) shared the target’s alphanumeric category. Therefore, the identity of the post-target distractor was confusable with the identity of the target. The sequence of events is illustrated in Fig. 1A. Manual responses were executed without time pressure at the end of each trial. While response screens varied across experiments, a shared feature of all the analyzed trials was that participants could report the post-target distractor, allowing DI responses.

All experiments included ten practice trials, which were not analyzed. While the different experiments contained a different number of trials, they all included at least 80 trials relevant for the current analysis. Therefore, we included the first 80 relevant trials from each experiment, resulting in the inclusion of 9520 experimental trials in total. Trials were coded based on whether participants committed a DI response or not.

#### Stimuli and design

Each trial began with the presentation of a fixation cross (a grey 0.2° x 0.2° “+” sign at the center of the screen). After 500 ms, two lateral RSVP streams, including 7 to 11 frames appeared along with the fixation cross. Each frame appeared for 50 ms, followed by an interstimulus interval (ISI) of 50 ms. The response display was a blank screen that remained present until a response was registered. Following this response, a blank screen appeared for 800 ms before a new trial started.

All stimuli in the RSVP streams were grey (CIE color coordinates: 0.309/.332, luminance 46.6 cd/m^2^). Each frame consisted of two alphanumeric characters appearing left and right of fixation. The characters were either 1° in size and appeared at a center-to-center distance of 3.5° from fixation (*n* = 64) or were 1.3° and appeared 4.5° from fixation (*n* = 55).

The target appeared with equal probability and unpredictably in one of few possible positions in the stream (5th to 8th), either in the left or right RSVP stream. This target frame contained one digit and one letter. The target appeared within the prespecified selection cue. In all the analyzed trials, the frame immediately preceding the target frame included two unreportable characters (i.e., a letter if the target was a digit, or a digit if the target was a letter) to prevent any pretarget intrusion errors. The earlier pretarget frames were equally likely to contain two unreportable characters or one reportable character and one unreportable character (with digit and letter location randomly selected for each frame). The length of the RSVP was determined by the target’s position, as the target frame was always followed by two additional frames. In all the analyzed trials, the frame immediately following the target contained a reportable character in the same location as the target. The final frame always included nonreportable characters.

### Results

#### Reliability of the full sample

We calculated two measures of reliability: Spearman–Brown split-half reliability (*r*’) and Cronbach’s alpha. After applying the Spearman–Brown correction formula, the split-half correlation of the intrusion scores for even and odd trials was *r’* (117) =.90. Cronbach’s alpha yielded a score of *α* =.89.

#### Iterative down-sampling

**Fig. 2:**
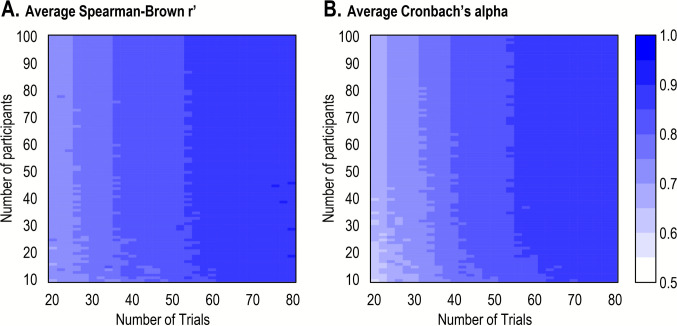
Average Spearman–Brown split-half reliability and Cronbach’s alpha as a function of the number of trials and the number of participants in Study 1. In each cell, average split-half reliability and Cronbach’s alpha were computed across 100 iterations for *t* trials (*x*-axis) and n participants (*y*-axis)

We investigated the number of participants and trials required to achieve acceptable levels of reliability (i.e., *r’* or *α* values higher than.80). Following Xu et al. (2018), we used an iterative down-sampling procedure, whereby we repeatedly sampled a random subset of trials and participants from the full dataset. The number of participants (*n*) varied from 10 to 100 and the number of trials (*t*) varied from 20 to 80 in steps of 2. For each of these combinations, we ran 100 sampling iterations. On each iteration, we randomly sampled *n* participants and *t* trials from the full dataset and calculated Spearman–Brown split-half reliability and Cronbach’s alpha. Finally, we calculated the average split-half reliability and Cronbach’s alpha for each combination of participants and trials. Figure 2 shows the results of the down-sampling procedure for both reliability estimates. This analysis revealed high average within-session reliability (*r’* >.80 and *α* >.80) even for a sample of 20 participants and 40–50 trials.

### Discussion

Study 1 revealed that a DI task produces very high levels of within-session reliability (*r*’ =.90, *α* =.89). Using a simulation, we could estimate that within-session reliability is high even with a small sample size and relatively few trials. For a sample size as small as *n* = 20, we can expect high average within-session reliability (*r*’ >.80 and *α* >.80) even for 40–50 trials. From a practical point of view, this suggests that researchers can get a reliable measure of DI rate with a 5-min session. In comparison, most attention measures produce lower reliability despite being much longer and requiring many more participants (Xu et al., 2018; Hedge et al., 2018a).

While encouraging, Study 1 has some limitations. Mainly, the analysis in Study 1 relied exclusively on a paradigm with two RSVP streams. This paradigm was developed mainly to facilitate N2pc research that necessitates lateral displays. In contrast, standard RSVP tasks traditionally employ a single RSVP stream, which results in fewer DI responses on average (Zivony & Eimer, 2023). Thus, it remains to be seen whether similar levels of reliability can be achieved when participants monitor only a single RSVP. Study 2 addresses this issue.

## Study 2

The first goal of Study 2 was to extend the results of Study 1 and examine within-session reliability in a task with a single RSVP stream, and to assess the between-session reliability of DI rates. We therefore invited participants to complete two identical experimental sessions. To preview our results, we found high within-session reliability but encountered surprisingly high attrition rates between the first and second sessions, preventing a strong conclusion on between-session reliability. We therefore re-examined this issue in Study 3.

The second goal of Study 2 was to examine the relationship between DI rates and individual differences in another task that measures temporal limitations in attentional processing, the attentional blink (AB). The AB is a nearly ubiquitous limitation in attending to two sequentially presented targets (Raymond et al., 1992). When two targets (*T1* and *T2*) are presented within the same RSVP stream, accuracy in reporting the second of the two targets is substantially reduced when it appears between 200 and 500 ms after the first (hereafter *the blink period*), relative to when it appears farther apart. Despite decades of research, some controversy remains regarding the exact causes of the AB. Nevertheless, it is widely agreed that the duration of the blink period is governed by the amount of time taken to encode T1 (Ouimet & Jolicœur, 2007; Visser, 2007) to working memory (WM). During this time, attentional processing and WM encoding of new targets are disrupted.

Whilst most of the research on the attentional blink has taken an experimental approach, some studies have documented consistent individual differences in sensitivity to the AB. In this line of research, an individual’s AB rate is often calculated as the difference between their performance in reporting the second target (T2) outside the blink period (e.g., when T1-T2 lag is seven items, or 700 ms) and during the peak of the blink period (e.g., when T1-T2 lag is three items, or 300 ms). Unfortunately, this line of research often produced inconsistent results (see Willems & Martens, 2016, for review). A possible reason for this is that, like other measures of attention, previous research into individual differences in AB rates has found inconsistent levels of reliability, ranging from.48 to.92 for both within-session and between-session reliability (Dale & Arnell, 2013; Dale et al., 2013). Another demonstration of this limitation is that individual differences in AB rates also vary with the exact variant of the AB task (e.g., Martens et al., 2010, 2015).

One approach to studying individual differences in AB performance focused on participants who consistently show no AB or extremely low AB rates (‘non-blinkers’) and compared them to participants with regular AB rates (blinkers). Two studies of non-blinkers (Martens et al., 2006; Troche & Rammsayer, 2013) have used the ERP method to measure the P3 component, a component that can be associated with WM encoding in the context of RSVP experiments (Hosseini et al., 2024). These studies found that non-blinkers produce earlier P3 components, which led to the conclusion that individual differences in AB performance reflect differences in the speed of WM encoding (Martens et al., 2006). That is, people who are quicker to encode T1 in WM show a smaller AB because T1’s encoding disrupts processing for a shorter period of time.

The association between the AB and the speed of WM encoding allows us to use this task to clarify the process reflected by individual differences in DI rates. We suggested that individual differences in DI rates reflect differences in the speed of attention. However, it is also possible that DI rates, similar to the AB, reflect individual differences in WM encoding speed. In other words, it is possible that participants with low intrusion rates are not quicker to attend to the target (and thereby extract the relevant sensory information from it) but are rather quicker to encode it (given the same amount of available sensory information). If that is the case, we should expect that DI rates should be strongly correlated with AB performance: participants who commit more DI errors should also show larger AB effects. In contrast, if DI rates uniquely reflect differences in the speed of attention (in line with previous accounts and findings, e.g., Broadbent & Broadbent, 1987; Chun, 1997; Zivony & Eimer, 2020, 2021), DI rates should be only weakly or not at all correlated with AB rates.

Only one study examined whether sensitivity to the AB is related to DIs, and found inconclusive results. Willems et al. (2013) compared blinkers and non-blinkers in an RSVP task in which the target was a colored letter among black letters, thereby allowing intrusions from all surrounding distractors. One of the measures employed in Willems et al. was the average reported position of the item relative to the target (see also Botella et al., 2001; Vul et al., 2008). With this measure, a correct response is indexed as + 0, whereas reporting the distractors that immediately precede or immediately follow the target is indexed as – 1 and + 1, respectively. Willems et al. were most concerned with performance during the blink period. For the purposes of the present study, the key results were those following T1 responses and following T2 outside the blink period, as these better represent participants’ tendency to report post-target distractors regardless of the AB. In one experiment, no difference was observed in the average reported position between blinkers and non-blinkers. In a second experiment with a larger sample size, an effect was observed only for T1, but its direction was not reported, though it can be speculated based on descriptive figures that non-blinkers showed more, not less, post-target DIs.

The results of Willems et al. (2013) suggest that AB and DI reflect two distinct phenomena. However, some aspects of their research prevent a clear conclusion at this point. First, Willems et al. used a unique color as the target-defining feature, which reduces the number of intrusions (Zivony & Eimer, 2021). Second, in their experiments, participants could report both pre-target and post-target distractors. While previous studies used the average reported position as a representation of a single process (Botella et al., 2001; Vul et al., 2008), it is likely that pre-target reports and post-target reports depend on separate attentional processes (Zivony & Eimer, 2023). For example, in a single-stream RSVP task, pre-target distractors may be reported because they benefit from (sustained) spatially focused attention, whereas post-target distractors may be reported because they are amplified by (transient) attentional engagement. In turn, this may reduce the reliability of Willems et al.’s (2013) measure of DIs. In contrast, our measure of DIs assesses only the perceptual competition between the target and immediately following post-target distractor, which has shown to result in stronger within-subject correlations (Zivony & Eimer, 2024a) and has been more closely associated with delays to the speed of transient attention (Zivony & Eimer, 2020; 2021). Therefore, we re-examined whether higher DI rates are associated with higher AB rates, using an RSVP task that isolates both measures. A strong correlation would support the conclusion that the two measures may reflect the same underlying mechanism, namely the speed of encoding.

Finally, and relatedly, we also conducted an additional control study to examine whether DI rates are associated with reading speed, a measure that is associated with AB magnitude (La Rocque & Visser, 2009). While DIs have been demonstrated using various types of stimuli (e.g., Adler & Intraub, 2021; Botella et al., 2001), our DI task relies on the identification of an alphanumeric stimulus and differentiating it from a following item from the same alphanumeric category. This raises the possibility that individual differences in reading speed, rather than in the speed of attention, explain DI rates. Study 2B was conducted to address this concern.

### Study 2A

#### Method

**Ethics.** All methods used in this study were approved by the institution’s ethical guidelines committee at the School of Psychology, University of Sheffield.

**Sample size selection.** Study 1 demonstrated that high within-session reliability can be achieved with a sample size of *N* = 25 and at least 50 trials. We hypothesized that if individual differences in intrusions and AB are caused by the same mechanism, then the correlation should be at least *r* =.40 (e.g., Arnell et al., 2006). A power analysis using G*Power indicated that 34 participants are required to detect such a correlation with 80% power and α =.05. Nevertheless, we sampled 64 participants, which would allow us to observe smaller correlations.

**Participants.** Participants consisted of 64 students from the University of Sheffield (47 women, 12 men, and five participants who did not report their gender, *M*_age_ = 19.3, *SD*_age_ = 2.2) who participated for course credits. All participants had normal or corrected-to-normal vision and were fluent in English.

**Apparatus.** The study was conducted using participants’ own computers. They downloaded and accessed the study via the E-Prime Go cloud service, and were instructed to sit approximately 60 cm from the screen (approximately an arm’s length), in a quiet and distraction-free environment. Manual responses were given through computer keyboards.

**Fig. 3:**
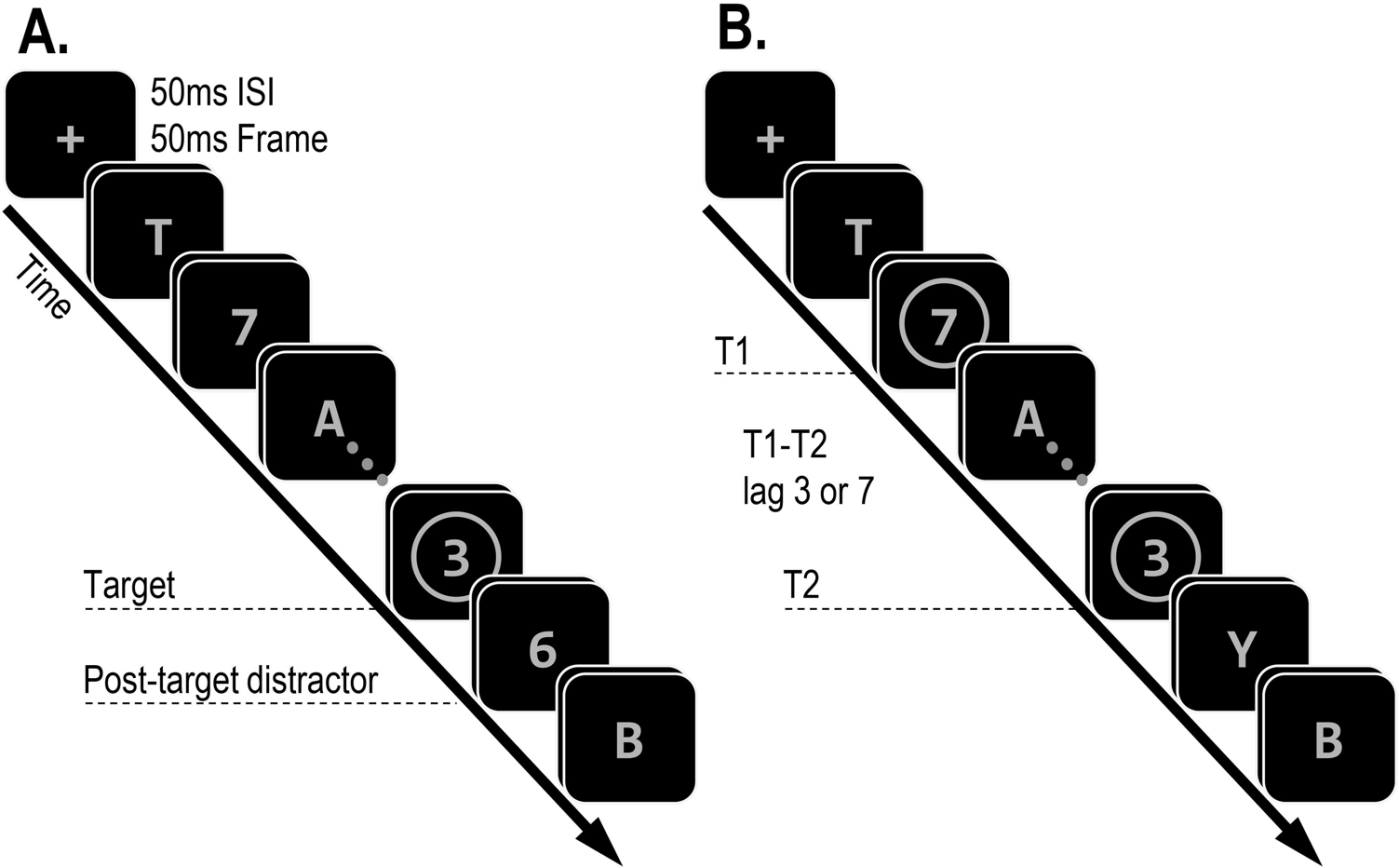
Illustration of the stimulus sequence in Study 2A. Participants had to identify either one or two digits that appeared inside a circle selection cue. On single-target trials (**A**), a single target digit appeared, followed by a reportable post-target distractor, allowing for DI responses. On two-target trials (**B**), two targets appeared (T1 and T2), either at lag 3 or 7 from each other. These targets were preceded and followed by nonreportable letter distractors, precluding DI responses

**Procedure.** The procedure was similar to the experiments reported in Study 1, except for the following differences. Participants entered a Qualtrics webpage where they were informed how to access the study. They provided consent and downloaded the experimental file to their private computer. All the stimuli were presented in a single RSVP stream, centered at fixation. The task (Fig. 3) was to identify either one or two digits (2–9) that were indicated by a circle (the selection cue). In the response screen, participants were informed whether there was a single target or two targets on that trial, and they provided the appropriate number of forced-choice responses.

The RSVP contained both letters and digits. On single-target trials, the target was always followed by a reportable digit distractor, allowing for DI responses. On two-target trials, both targets were followed by nonreportable letter distractors and so DI responses were not possible. In these trials, the two targets were either presented at a target-to-target lag of three frames (lag 3) or seven frames (lag 7). Thus, T2 was either inside the blink period (lag 3) or outside of it (lag 7).

In the first session, participants completed ten practice trials, followed by four blocks of 30 experimental trials (a total of 120 trials). Half of the trials were single-target trials and half were two-target trials. On two-target trials, the second target randomly appeared at either lag 3 or lag 7. A week after their participation, participants were contacted and were requested to complete a second session. Out of the full sample, only 40.6% (*n* = 26) agreed to participate in the second session. The procedure, stimulus, and design of the second session were identical to those of the first.

**Stimulus and design.** The stimuli were the same as those used in Study 1, except for the following differences. Each trial began with a fixation cross in the center of the screen and was displayed for 500 ms. After this time, the fixation cross was replaced by a single RSVP stream. On single-target trials, the target was presented in either the 12th, 14th, or 16th frame of the stream. On two-target trials, the position of the second target (T2) was either the 12th, 14th, or 16th frame, and the first target (T1) appeared either three or seven frames prior to it. The stream ended two frames after the last target. Following (one or two) responses, there was a blank display for 500 ms before the study automatically moved on to the next trial.

Half of the distractor stimuli in the stream were letters and half were digits, which were randomly selected without repetition from the set of possible digits (2–9) and the English alphabet (except for the letters I and O). Targets were also selected without repetition from the set of possible digits. The order of the stimuli in the stream was random except for the possible restrictions: on single-target trials, the target was preceded by a letter and followed by a digit; on two-target trials, both targets were preceded and followed by letters; the last frame always contained letters.

All the stimuli in the stream were grey (RGB: 125, 125, 125). Since participants completed the task on their personal computer, the exact luminosity of the stimuli could not be determined. Letters and digits were 1.3° in height (assuming a viewing distance of 60 cm). The target-defining circle cues were 1.68° in diameter. The response screen contained all possible digits, presented on the middle third of the participants’ display, with each digit appearing in rising order and equidistant from one another.

**Data curation and analysis**. Practice trials were not analyzed. Study 2A did not employ any continuous measures, and therefore no individual trials were considered to be outliers. On the first session, one participant had an intrusion rate that was higher than 3 SDs from the mean intrusion rate (93.3% versus *M* = 29.6%, *SD* = 20.1%) and one participant had T1 accuracy rate that was lower than 3 SDs from the mean T1 accuracy rate (23.3% versus *M* = 82.9%, SD = 14.7%). Therefore, for correlational analyses involving these measures, we excluded these participants to prevent undue influence of these extreme results on the observed correlations.

In this and the following studies, we conducted standard frequentist analyses (e.g., correlation, repeated measures ANOVA) alongside comparable Bayesian analyses. Specifically, we calculated Bayes Factors in favor of the alternate hypotheses (*BF*_*10*_) and in favor of the null hypothesis (*BF*_*01*_). Which BF was reported was based on whether the frequentist test yielded a significant result (*p <.05*) or not. The inclusion of BFs is particularly helpful in cases where the frequentist analysis does not yield a significant result, where a BF can provide positive evidence in favor of the null hypothesis (BF_01_). Specifically, BFs provide a measure of the degree to which our beliefs should be updated given the data. For example, a *BF* of 3 means that, given the data, we should update our belief (relative to our prior belief) in favor of the supported hypothesis by a factor of 3. Following Dienes and Mclatchie (2018), we consider a *BF* to provide substantial evidence for the related hypothesis if it is larger than 3. We consider Bayes factors smaller than 3 to provide inconclusive evidence.

For correlation analyses, we calculated *BF*s using JASP (0.18.0.3). Since we had a priori expectations regarding the direction of correlations, but not their exact effect sizes, we used directional BFs and the default JASP prior (stretched beta prior of 1.0). However, in all cases, we reached the same conclusions (i.e., substantial evidence or inconclusive evidence) whether we used a narrower or wider prior (0.5 and 1.5). For ANOVAs and *t* tests, we conducted BF analyses using the anovaBF and lmBF functions from the BayesFactor package in R (Morey et al., 2018). As recommended by Van Doorn et al. (2023), we used the “maximal” model (i.e., the model that includes both participant intercepts and effect slopes as random effects) to evaluate our effects, although all the results were comparable when only participant intercepts were included as random factors. Bayes factors associated with a two-way interaction were calculated by dividing two Bayes factors: (*i*) the Bayes factor associated with the main effect for both factors and the interaction term, and (*ii*) the Bayes factor associated with the model that includes only the two main effects. Since we had no a priori expectations regarding these effects, we used the default medium prior (*r* =.50), yet in all studies, we reached the same conclusions with wider priors (*r* =.707 or *r* = 1.0).

### Results

**Session 1.** On two-target trials, accuracy in reporting T1 was generally high (*M* = 82.9%, *SD* = 14.8%), as was accuracy in reporting T2 when it was outside the blink period (*M* = 81.1%, *SD* = 17.7%). T2 accuracy was substantially lower when T2 appeared inside the blink period (*M* = 40.8%, *SD* = 20.0%), and this difference was significant, *t*(63) = 16.63, *p* <.001, *d* = 2.08, *BF*_*10*_ > 100. AB rate was calculated as the difference between these latter two measures (*M* = 40.3%, *SD* = 19.4%). When a single target appeared in the stream followed by a reportable distractor, accuracy was *M* = 62.3% (*SD* = 23.1%), and intrusion rates were *M* = 29.6% (*SD* = 20.0%).

**Session 2.** Results from participants who completed the second session (*n* = 26) were similar to those observed in the first session. T1 accuracy was *M* = 86.5% (*SD* = 13.0%) and the size of the AB was *M* = 37.7% (*SD* = 19.4%). The difference in these measures between the two sessions was not significant, both *t*s < 1, *BF*_*01*_*s* > 16. The average intrusion rate was 26.8% (*SD* = 20.4%), which was also not significantly different from the first session, *t*(25) = 1.10, *p* =.28, *d* = 0.22, *BF*_*01*_ = 5.38.

**Within-session reliability.** We calculated the Spearman–Brown split-half reliability for the three main measures: T1 accuracy, AB rate, and DI rates, on both the first and second sessions. Within-session reliability was high for T1 accuracy, *r’* =.94 and *r’* =.90 (for the first and second sessions, respectively), lower for the AB, *r’* =.62 and *r’* =.79, and high for DI rates, *r’* =.94 and *r’* =.96.

**Fig. 4:**
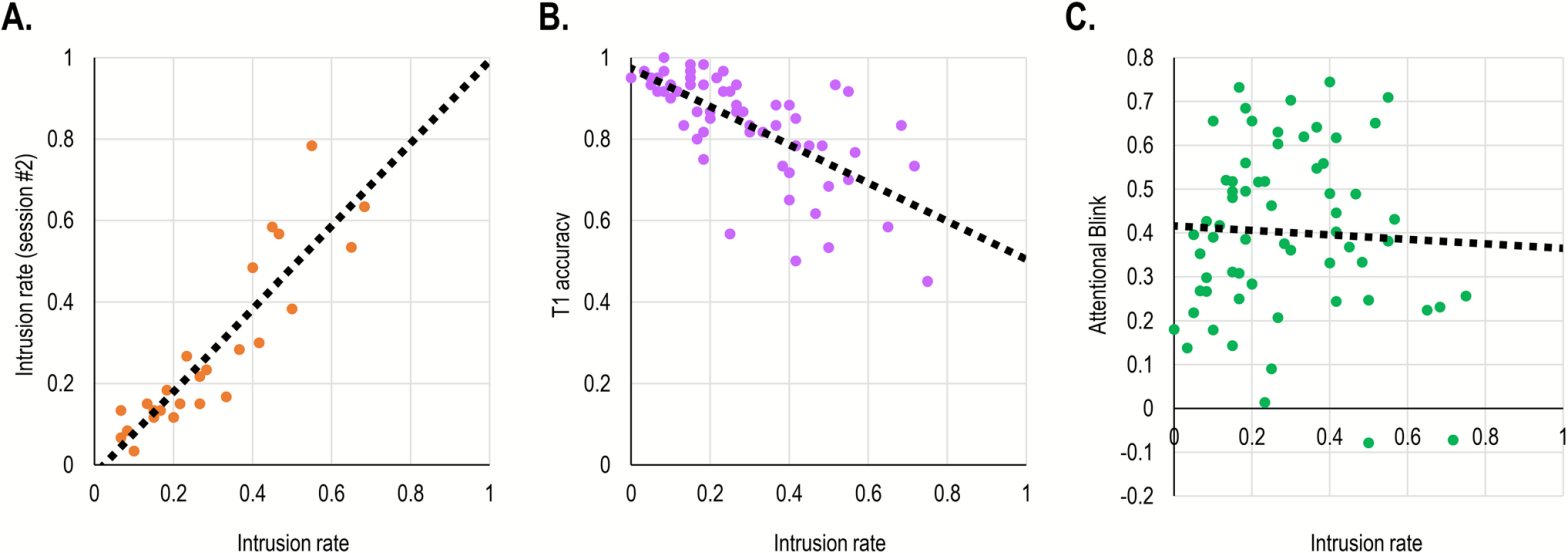
Scatterplots describing the relationship between DI rates (*x*-axis) and: **A** DI rates on a second session, **B** T1 accuracy, and **C** attentional blink (T2 accuracy at lag 7 minus accuracy at lag 3) in Study 2A. The *dotted line* reflects the linear regression equation calculated based on these results

**Between-session reliability.** We calculated the between-session reliability as the correlation between the three main measures. The correlation was significant for all three measures. It was strongest for DI rates, *r*(24) =.90, *p* <.001, *BF*_*10*_ > 100, (Fig. 4A), weaker for T1 accuracy, *r*(24) =.72, *p* <.001, *BF*_*10*_ > 100, and weaker still for the AB, *r*(24) =.61, *p* <.001, *BF*_*10*_ > 100.

**Correlations between measures.** Correlations between the three measures were calculated only for the first session. The only significant correlation was between intrusion rates and T1 accuracy, *r*(60) = –.68, *p* <.001, *BF*_*10*_ > 100: participants with higher intrusion rates also had lower T1 accuracy (Fig. 4B). Importantly, the correlation between intrusion rates and AB rates was non-significant, *r*(62) = –.05, *p* =.96, *BF*_*01*_ = 5.93 (Fig. 4C). Finally, the correlation between AB rates and T1 accuracy was also non-significant, *r*(61) =.21, *p* =.09, but the evidence in favor of this null effect was inconclusive, *BF*_*01*_ = 1.65.

### Study 2B

#### Method

**Ethics.** All methods used in this study were approved by the institution’s ethical guidelines committee at the School of Psychology and Clinical Language Sciences, University of Reading.

**Participants.** The participants consisted of 40 students from the University of Reading (31 women, eight men, one non-binary, *M*_age_ = 20.3, *SD*_age_ = 2.8) who participated for course credits. All participants had normal or corrected-to-normal vision and were fluent in English.

**Apparatus.** The study was conducted in one of three labs with a Viglen desktop PC and DELL standard screen (100 Hz; 1920 x 1080 screen resolution), with participant viewing distance at approximately 60 cm. Manual responses were registered via a standard computer keyboard. Luminance was not measured.

**Procedure.** Participants provided written informed consent via an online MS Form prior to attendance in the lab. They completed the Test of Word Reading Efficiency Second Edition (TOWRE-2; Torgesen et al., 2012), whereby they were asked to read aloud as many single words (Single Word Efficiency, SWE) and pseudowords (Phonemic Decoding Efficiency, PDE) of increasing difficulty as accurately and as quickly as possible from printed lists of 108 and 66 words respectively, within 45s. The measure yielded by each task reflects the number of correct words (SWE) and pseudowords (PDE) produced by participants within the fixed timespan. Participants then completed the DI task.

**Stimuli and design.** The stimuli and design of the DI task were the same as the stimuli and design described in Study 2A, except for the following changes. Participants completed a single session with 10 practice trials and 60 experimental trials, presented in two blocks. There was only a single target presented in the RSVP, and therefore participants provided only one response per trial. The target was always followed by a reportable distractor. The target appeared in either the 8th, 10th, or 12th frame.

**Data curation.** Practice trials were not analyzed. Study 2B did not employ any continuous measures, and therefore no individual trials were considered to be outliers. No participant had a score more extreme than 3 SD above or below the mean of any measure, and therefore no participants were treated as outliers.

#### Results

**Fig. 5.**
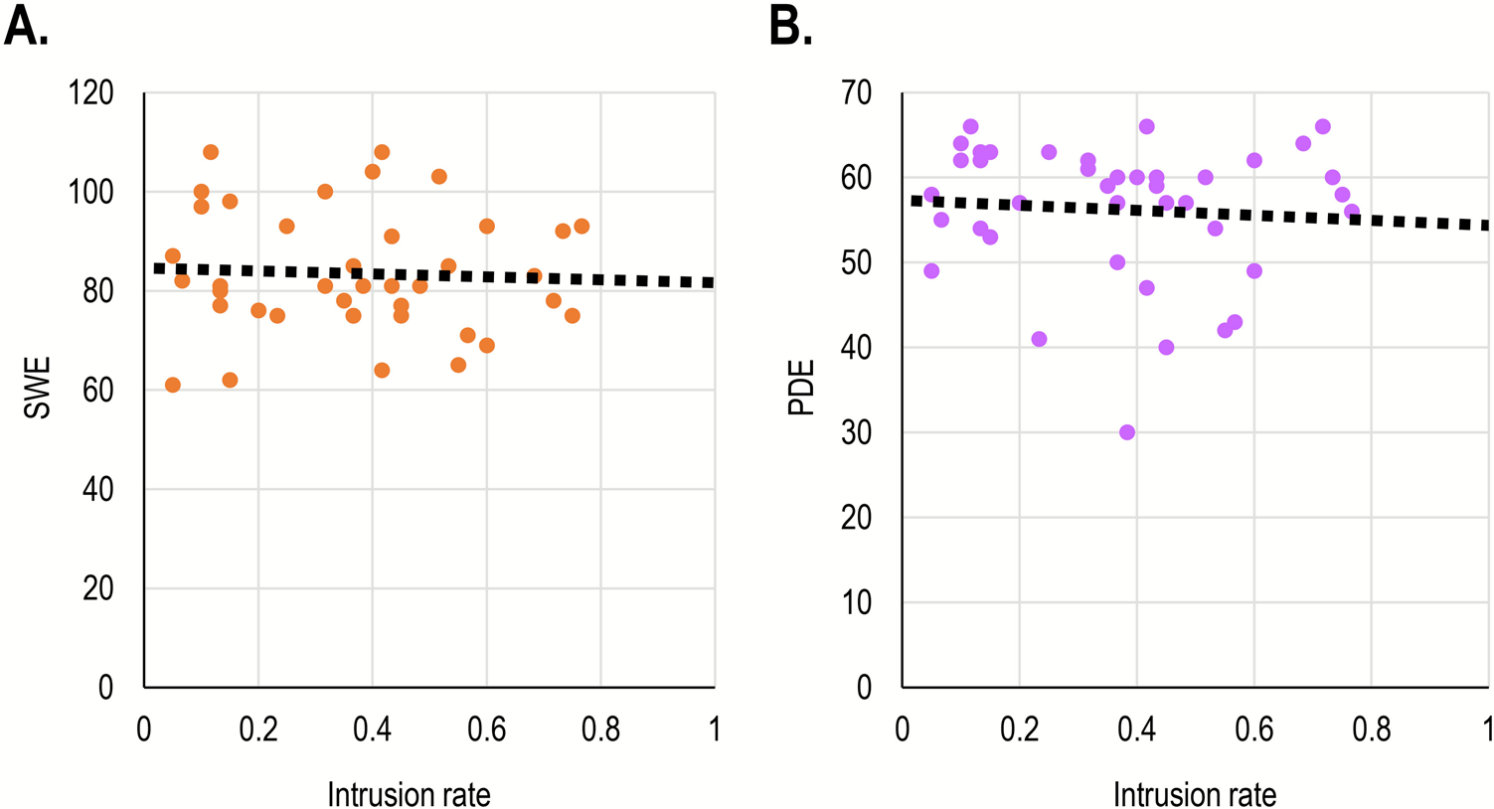
Scatterplots describing the relationship between DI rates (*x*-axis) on the one hand and (**A**) SWE, and (**B**) PDE, on the other, in Study 2B. The *dotted line* reflects the linear regression equation calculated based on these results

We calculated the correlation between intrusion rates, SWE, and PDE. As expected, SWE and PDE were significantly correlated, *r*(38) =.53, *p* <.001, *BF*_*10*_ > 100. In contrast, there was nearly no association between intrusion rates and SWE, *r*(38) = –.05, *p* =.76, *BF*_*01*_ = 4.85, nor between intrusion rates and PDE, *r*(38) = –.08, *p* =.64, *BF*_*01*_ = 4.56 (Fig. 5).

#### Discussion

Study 2 produced several clear-cut results. First, like Study 1, DI rates had high within-session reliability (*r*’ >.90), even with a small sample and relatively few trials. Within-session reliability was also very high for accuracy in reporting the first target (T1) on two-target trials, but was lower for AB rates. Second, DI rates showed high between-session reliability, which was higher than the between-session reliability of either T1 accuracy or AB rates. These findings are consistent with previous AB studies that revealed moderate levels of reliability for AB rates (e.g., Dale et al., 2013). While the observed between-session reliability of DI rates is promising, this finding comes with the caveat that a high proportion of our participants did not complete the second session. It is therefore possible that self-selection played a role in producing high between-session reliability. We therefore revisited test–retest reliability in Study 3.

The third finding was that participants’ DI rates were negatively associated with T1 accuracy but were unrelated to the AB effect. We did not make explicit a priori predictions about DI rates and T1 accuracy. Since T1 accuracy is often very high, we did not expect a high rate of between-subject variability in this measure. Nevertheless, the strong negative correlation between these measures may not be surprising. In RSVP, a target’s identity is at risk of being overridden by the following item. While this risk is greater when the post-target item is reportable (see Fig. 1B), it can also occur if the post-target item is completely irrelevant to the task. Thus, individual differences in the speed of attention should affect the likelihood of reporting the target even when DI responses are not possible. Note, however, that we do not advocate using T1 accuracy as a measure of individual differences in the speed of attention. First, in the absence of competition from a nearby distractor, target reports should be more resilient to changes in the speed of attention. Second, under such conditions, target reports are more likely to reach ceiling levels (as has been demonstrated by numerous AB studies), which consequently reduces the variability of this measure and, thereby, reduces its reliability (Hedge et al., 2018a).

The lack of association between intrusion rates and AB rates suggests that they do not reflect the same mechanism. On the face of it, the two phenomena seem closely related, as the AB occurs due to (amongst other things) a disruption to attentional processes (Nieuwenstein, 2006; Zivony et al., 2018). However, people who inherently show delayed attentional engagement (as demonstrated by DIs) are not more necessarily sensitive to additional disruption caused by the AB’s deleterious effect. One possible limitation of this conclusion is that we could not compare DI rates between blinkers and non-blinkers, as has been done in previous studies. Indeed, in Study 2A, only 3 (out of 64) participants in our sample could be classified as non-blinkers. Therefore, it is possible that in a paradigm that can more clearly distinguish between blinkers and non-blinkers (e.g., Willems et al., 2013), a correlation between the two measures could emerge. Nevertheless, we suggest that the results allow us to reject the notion that the two measures reflect the same underlying mechanism. Had that been the case, a strong correlation should have been observed, despite the small number of non-blinkers. Therefore, we conclude that the two phenomena reflect two separate limitations to temporal selection that are functionally independent. While individual differences in AB performance reflect differences in the speed of WM encoding, individual differences in DI rates are more closely linked to attentional deployment.

Finally, our control Study 2B revealed no correlation between DI rates and reading efficiency, and this conclusion was supported by a Bayesian analysis. The small sample size in the current study precludes any strong conclusions about the existence or the absence of a relationship between the speed of attention and reading efficiency. However, they do suggest that DI rates are not entirely caused by differences in reading efficiency, which would have resulted in a strong correlation.

Thus, taken together, the findings of Studies 2A and 2B suggest that individual differences in DI rates are not merely a reflection of variability in the same mechanisms underpinning the AB effect or reading efficiency. This conclusion not only helps identify the exact processes reflected in DI rates but also has implications for future research. Had individual differences in AB effects and DI rates been closely linked, one could have relied on the vast AB literature to predict the factors that may affect DI rates. However, since the AB and DI tasks measure different constructs and two different temporal limitations on selection, learning about one is not necessarily informative about the other.

## Study 3

The purpose of Study 3 was twofold. The first goal was to re-examine within-session and between-session reliability of DI rates in a larger sample (Study 3A) and after a much longer period – 1 year – between test and retest sessions (Study 3B). The second goal was to further examine the convergent and divergent validity of DI rates.

First, if DI rates reflect individual differences in the speed of attention, then they should correlate with other measures associated with that speed. In Study 3A, we examine the correlation between DI rates and RTs in standard attention tasks, such as the Visual Search task. The speed of attention reflects only one of the multiple processes that affect overall RTs (Palmer et al., 2011). Nevertheless, barring a negative correlation between these processes, variability in the speed of attention should predict individual RTs to some degree. Therefore, if DI rates assess the speed of attention, they should also be associated with slower RTs. In Study 3B, we examine the correlation between DI rates and performance on a Time Judgment task. The Time Judgment task required participants to observe a moving clock and indicate the position of a clock hand at a cued moment. The original version of this task was used by Wundt more than a century ago (Wundt, 1883). More recently, Carlson and colleagues (2006) showed that (similar to DI rates) manipulations that slow attentional engagement also result in delayed time judgment relative to the actual cued time. They concluded that errors in time judgment (hereafter time errors) can be used to directly measure the speed of attention. Therefore, if DI rates assess the speed of attention, they should also be associated with larger time errors.

Second, we examined whether DI rates correlate with individual differences in measures of attentional control, which we define as the ability to ignore irrelevant information or resolve different kinds of conflicts (von Bastian et al., 2020). While individuals’ attentional control has been shown to be predictive of performance in other cognitive tasks (Hedge et al., 2020), it should be largely irrelevant for performance in a task that measures the speed of attention. Moreover, a standard DI task demands only little attentional control, as there are few conflicts to be monitored. The goal in a standard DI task is quite simple: Participants are required to attend to a selection cue, which is both salient and unique. In our variant of the task, the selection cue is the only circle presented in the RSVP, and also the only stimulus that is not masked by a preceding or following stimulus. Therefore, attending to the cue relies on both an easily maintainable top-down attentional template for a specific and simple feature (the circle), as well as automatic saliency detection mechanisms (see Luck et al., 2021). These low demands of attentional control should leave little room for individual differences, and accordingly, little room for correlations between attentional control and DI rates.

**Fig. 6:**
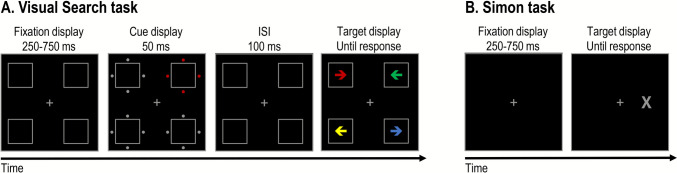
Sample sequence of events in the Visual Search (**A**) and Simon tasks (**B**) used in Study 3A. In the visual search task, participants had to report whether the *red arrow* was pointing left or right. Prior to the target display, non-predictive cues consisting of four refd dots randomly appeared around one of the squares. In this example, the target appeared in a different location than the cue. In the Simon task, participants had to report whether the letter in the target display was an “X” or an “O”

To test the association between DI rates, overall RT, and attention control, in Study 3A we included two attentional control tasks, the Simon task and a Visual Search/Cueing task (Fig. 6). In the Simon task (Simon, 1990), participants are presented with a stimulus that appears either on the left or right side of the monitor and have to respond to the target’s identity either with their left or right hand (Fig. 6B). While the stimulus location is irrelevant to the task, most participants find it difficult to ignore this information. The automatic association between the stimulus location and the motor response creates a conflict that results in lower accuracy and longer response times (the Simon effect). Some participants are highly efficient in maintaining the task goal and ignoring or resolving irrelevant conflicts, and these participants should have a smaller Simon effect (e.g., Hedge et al., 2020). In the Cueing task, participants perform a simple Visual Search task. However, prior to the Visual Search display, an irrelevant and non-predictive distractor (cue) appears (Fig. 6A). Such cues are known to involuntarily capture observer’s attention (Folk et al., 1992; Luck et al., 2021). When spatial attention is captured to an irrelevant location, it needs to reorient to the target location before attentional engagement can occur. Therefore, RTs should be slower when the cue appears in the location of a non-target relative to when it appears in the target location (a cueing location effect). Participants’ ability to resist attentional capture has been shown to be predictive of performance in cognitive tasks (Fukuda & Vogel, 2011). Since it is more closely related to the kind of attentional control that may affect performance in the DI task, we used participants’ location effects as a second measure of attentional control. A weak or no correlation between DI rates and attentional control measures would be compatible with the notion that individual differences in DI rates reflect variability in the speed of attention, rather than differences in attentional control.

### Study 3A

#### Method

All methods used in this study were approved by the institution’s ethical guidelines committee at the School of Psychology, University of Sheffield.

**Sample size selection.** We aimed to sample 100 participants to achieve 80% power to detect effects of *r* =.25 (when α =.05), which is what we expected to be the effect size of the correlation between an error-based measure (i.e., DI rates) and an RT-based measure (i.e., overall RT in the Visual Search and Simon tasks) that share an underlying process (Hedge et al., 2018a,b).

**Participants.** Participants were recruited via Prolific. Overall, 101 participants completed the first session (*M*_age_ = 34.8 years, *SD*_age_ = 6.9), of whom 53 were men, 46 were women, and two participants who did not report their gender identity (other gender identities were available for report but these options were not selected). They were paid £3 for each session. Out of the original pool of participants who completed the first session, 100 participants agreed to complete the second session. However, due to technical problems, five participants could not complete the second session. The results of these participants were included for all analyses that pertained to the first session.

**Procedure.** On each session, participants completed a DI task and either the Visual Search task or the Simon task. Within each session, the order of the two tasks was randomized. Fifty-one participants completed the Visual Search (and DI) task on the first session, and the rest completed the Simon (and DI) task on the first session. To account for variability caused by presentation order, we replicated all the analyses after using the correction applied by Wilhelm and Oberauer (2006) and by Von Bastian and Oberauer (2013). This correction removes the average group difference (e.g., Simon task session first versus Visual Search task session first) from one group’s score, thereby equalizing between the two and removing any order-related variance. All the conclusions (i.e., significant versus non-significant, substantial evidence versus inconclusive evidence) were the same after these corrections. Moreover, preliminary analysis indicated that the order of sessions in which participants completed the Visual Search and Simon tasks (first or second), as well as the order of tasks within each session (DI task first or the alternative task first) did not affect RTs and accuracy rates. Therefore, we collapsed the data across the different viewing order conditions, and we report the analyses on the original data.

Like Study 2B, the DI task included ten practice trials and 60 experimental trials, presented in 30-trial blocks. The Visual Search task and the Simon task each included 40 practice trials and 200 experimental trials presented in 50-trial blocks. Participants were allowed a self-paced rest between blocks. During these breaks, participants were informed of their average accuracy and RT for the preceding block in both the Visual Search and Simon tasks.

#### Stimuli and design

***Distractor intrusion task.*** The stimuli and design were the same as those described in Study 2B.

***Visual Search (Cuing) task.*** The sequence of events on each trial is presented in Fig. 6A. Participants were instructed to report as quickly and as accurately as possible whether a red arrow was aiming right or left by pressing the “K” key with their right hand or the “A” key with their left hand, respectively. These stimuli were chosen to minimize inaccurate responses due to weak stimulus-response associations and due to individual differences in response selection efficacy. Each trial began with the fixation display, which appeared for a random duration ranging from 250 ms to 750 ms. Then, on 80% of the trials, a cue display appeared for 50 ms. The cue display contained one set of four red dots, which appeared randomly in one of the four locations.

When present, the cue display was followed by the fixation display for an additional 100 ms and then by the target display, which remained on the screen until the response. Participants were asked to respond as quickly as possible and to aim for responses faster than 800 ms with an accuracy above 90%. Errors were followed by a display where the fixation was replaced by “X” for 100 ms. After the response, a blank screen appeared for 500 ms, after which a new trial began. Participants were instructed to maintain their eyes on the fixation cross. They were informed about the presence of the cues, which were not informative of the target’s location, and were instructed to ignore them.

Stimuli were four 0.8° × 0.5° arrows, drawn with 4-pixel-thick lines, which appeared against a black background. The fixation display consisted of a 0.2° × 0.2° cross in the center of the screen, surrounded by four 1.5° × 1.5° empty square placeholders, drawn with 1-pixel-thick lines. These squares appeared at the corners of an imaginary 3° × 3° square centered at fixation. The cue and target displays were similar to the fixation display except for the following differences. In the cue display, four filled dots (0.2° in diameter) appeared at cardinal locations around all of the placeholders, with dot-placeholder center-to-center distance set at 1.1°. One set of dots was red (RGB: 220, 0, 35), and the rest were grey (125, 125, 125). In the target display, right-pointing or left-pointing stimuli were presented in the center of each placeholder. One arrow, the target, was red (220, 0, 35), and the three distractors were yellow (103, 102, 0), green (0, 115, 0), or blue (71, 71, 250). The target display always contained two left-pointing arrows and two right-pointing arrows.

A cue display was present on 80% of the trials and absent on 20% of the trials. On trials that included a cue, the cue and target locations were randomly set on each trial. Accordingly, the cue and target appeared at the same location on ~ 20% of the trials (same location trials), and on one of the different locations on ~ 60% of the trials (different location trials), making the cue unpredictive of the target’s location.

Note that since the cue shared the target’s color, it should result in contingent capture (Folk et al., 1992), rather than pure bottom-up capture. We chose this variant of a cueing task because previous studies have shown that this type of cueing effect was more robust (i.e., it occurred in more participants) and more reliable than pure bottom-up cueing effects (Roque et al., 2016). Nevertheless, Roque et al. found that contingent capture is correlated with bottom-up capture.

***Simon task.*** The sequence of events on each trial is presented in Fig. 6B. Participants were instructed to report as quickly and as accurately as possible whether a single object was the letter “X” or “O”, by pressing the “K” (the right-hand key) or “A” (left-hand key) keys. The association between key and letter was counterbalanced between participants. Each trial began with the fixation display, which appeared for a random duration ranging from 250 ms to 750 ms. Then, the target letter appeared either to the left or right of fixation until the response. Participants were asked to respond as quickly as possible and aim for responses faster than 800 ms with an accuracy of above 90%. Errors were followed by a display where the fixation cross turned red for 100 ms. After the response, a blank screen appeared for 500 ms, after which a new trial began. The target appeared on the same side as the associated response key (compatible condition) on 75% of the trials and the side associated with the alternative response (incompatible condition) on the rest. For example, if “X” was associated with the left-hand key (and “O” with the right-hand key), it appeared left of fixation on 75% of the trials and right of fixation on 25% of the trials. This version of the Simon task was used because previous studies showed that it increases reliability relative to tasks where compatible and incompatible trials were equiprobable (Borgmann et al., 2007). The targets were written in “Consolas” font, were 1° in height, grey (125, 125, 125), and their center-to-center distance from fixation was 3°.

***Data curation and analysis.*** Practice trials were not analyzed. For the DI task, all other trials were analyzed, and none of the participants were removed, as none showed an intrusion rate more extreme than 3 SDs from the mean intrusion rate. For the Visual Search and Simon tasks, different sets of trials were analyzed depending on the analysis. For analyses of accuracy rates, all trials were analyzed, regardless of RTs. For RT analysis, only correct trials were analyzed (95.5% of trials for both Visual Search and Simon tasks). For both the Visual Search task and Simon task, trials were excluded from analysis if they were faster than 150 ms or slower than 1000 ms, resulting in the removal of 0.1% of trials. Next, trials were considered to be outliers (and were excluded from analysis) if they were either slower or faster by 3 SDs than a participant’s average RT on that specific condition. This analysis resulted in the exclusion of 0.7% and 1.4% of trials in the Visual Search and Simon task, respectively. Finally, following this procedure, participants were considered to be outliers if their mean RTs were slower or faster than 3 SDs from the average RT in either task. One participant had a mean RT of 733 ms in the Visual Search task, compared to a mean RT of 547 (*SD* = 58), and another had a mean RT of 594 ms in the Simon task, compared to a mean RT of 436 ms (*SD* = 52). The results of these participants were excluded only from the analyses related to the particular tasks for which they were outliers. This was done to reduce their undue effect on any correlational analysis without completely rejecting all their results.

Other than overall reaction time, the introduction of a spatial cue in the Visual Search task allowed us to examine cueing effects. Cueing effects were examined by entering RTs and accuracy rates to repeated-measures ANOVA with cue condition (absent, different location, and same location) as an independent factor, followed by Bonferroni-corrected post hoc comparisons. The Simon effect was examined by using a dependent-samples *t* test where the compatible condition is compared to the incompatible condition, and RTs and accuracy rates were used as dependent variables.

Within-session reliability was examined by calculating Spearman–Brown split-half reliability. This measure was calculated for overall RTs in the Visual Search task and the Simon task, the cueing effect on RTs in the Visual Search task, and the Simon effect on RTs. Split-half reliability was also calculated for intrusion rates, separately for the first session and the second session. Between-session reliability was examined only for DI rates (as it was the only task that repeated on both sessions) by calculating the Pearson correlation between DI rates.

#### Results

**Cueing.** The presence and the location of the cue affected RTs, *F*(2, 192) = 572.20, *p* <.001, *η*^*2*^_*p*_ =.86, *BF*_*10*_ > 100. RTs were slowest when the cue appeared in a different location than the target (*M* = 568 ms, *SD* = 56), faster when the cue was absent (*M* = 531 ms, *SD* = 57), and faster still when the cue appeared in the location of the target (*M* = 495 ms, *SD* = 55). Post-hoc tests indicated that the comparison between each pair of conditions was significant, all *p*s <.001, *BF*_*10*_*s* > 100. Analysis of accuracy indicated that the location of the cue, but not its presence, affected accuracy. The difference between all three conditions was significant, *F*(2, 194) = 31.62, *p* <.001, ${\eta }_{p}^{2}$ =.24, *BF*_*10*_ > 100. Accuracy was highest when the cue appeared in the location of the target (*M* = 97.8%, *SD* = 4.0%), slightly lower when the cue was absent (*M* = 97.5%, *SD* = 3.6%), and lower when the cue appeared in a non-target location (*M* = 95.0%, *SD* = 3.7%). Accuracy under the non-target cue location condition was significantly lower than when the cue was absent or appeared in the target location (both *p*s <.001, *BF*_*10*_*s* > 100), whereas the difference between accuracy under the no-cue and same-location cue conditions was not significant (*p* >.05, *BF*_*01*_ = 26.31).

**Simon effects.** Simon effects emerged for both RTs and accuracy. Responses were slower and accuracy rates were lower when the target appeared in the response-incompatible location than the response-compatible location, *M* = 497 ms (*SD* = 59) vs. *M* = 416 ms (*SD* = 48), *t*(97) = 28.22, *p* <.001, *dz* = 2.85, *BF*_*10*_ > 100, and *M* = 85.7% (*SD* = 9.5%) vs. *M* = 98.9% (*SD* = 1.8%), *t*(98) = 14.65, *p* <.001, *dz* = 1.47, *BF*_*10*_ > 100, respectively.

**Reliability.** Split-half reliability was very high for overall RT in both the Visual Search and Simon tasks, *r’* =.99 and *r’* =.98, respectively. Split-half reliability was lower for the cueing effects and Simon effects, *r’* =.58 and *r’* =.66, respectively. In contrast, split-half reliability was high for intrusion rates on both the first and second session, *r’* =.95 and *r’* =.94. Importantly, test–retest reliability for intrusion rates was high, *r*(94) =.90, *p* <.001, *BF*_*10*_ > 100.

**Fig. 7:**
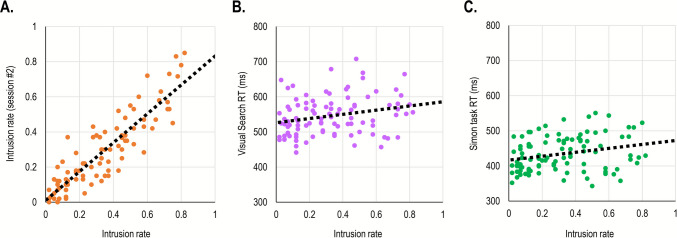
Scatterplots describing the relationship between measures in Study 3. The *x*-axis represents DI rates in a first session and the *y*-axis represents (**A**) DI rates on a second session, (**B**) overall RT in a visual search task, and (**C**) overall RT in a Simon task. The *dotted line* reflects the linear regression equation calculated based on these data

**Correlations.** For this analysis, only intrusion rates from the first session were used (although the results were comparable if an average of both sessions was used instead). A Pearson correlation was computed to examine whether intrusion rates correlated significantly with overall RTs, cueing effects, and Simon effects. This analysis revealed a significant correlation with Visual Search RTs, *r*(95) =.25, *p* =.015, *BF*_*10*_ = 3.73 (Fig. 7A), and Simon task RTs, and *r*(97) =.26, *p* =.01, *BF*_*10*_ = 4.98 (Fig. 7B). As can be seen, there was only a marginal difference between these correlations. This observation was confirmed by a non-significant Fisher* r*-to-*z* transformation test, *Z* =.07, *p* =.95. Finally, there was no correlation between DI rates and either cueing effect, *r*(97) = –.05, *p* =.60, *BF*_*01*_ = 8.85, or the Simon effect, *r*(98) =.17, *p* =.10, *BF*_*01*_ = 2.65 respectively, although evidence for the latter was inconclusive.

**Accounting for age.** One factor that is well known to affect overall RTs is general slowing caused due to participants’ aging. Indeed, in the current study, despite the limited range of participants’ age (18–45), there was a significant positive correlation between age and overall RT in both the Visual Search task and the Simon task, *r*(93) =.36, *p* <.001, *BF*_*10*_ > 100, and *r*(96) =.25, *p* =.014, *BF*_*10*_ = 4.05. Therefore, as an exploratory analysis, we examined whether the relationship between DI rates and overall RTs in the Visual Search and Simon tasks remained significant when age is controlled for. To do so, we recalculated the correlations between DI rates and overall RTs, while controlling for age (i.e., when age is partialed out). For this analysis, participants who did not report their age (*n* = 2) were excluded. This analysis indicated that the correlations between intrusion rates and overall RTs for both the Visual Search and Simon task remained significant, r_partial_(93) =.27, *p* =.008, and *r*_partial_ (94) =.28, *p* =.006. Indeed, the correlation coefficients were slightly larger compared to when age was not accounted for, suggestive of a slight suppressive relationship between age and the DI-RT association.

### Study 3B

#### Method

**Sample size selection.** This study had two goals. The first was to examine test–retest reliability after 1 year. The second was to examine the association between DI rates and time errors in a Time Judgment task, thought to reflect variability in the speed of attention. Like Study 2A, we hypothesized that if individual differences in intrusions and time errors were due to the same mechanism (speed of attention), then the correlation should be at least *r* =.40 (e.g., Arnell et al., 2006). A power analysis using G*Power indicated that 34 participants are required to detect such a correlation with 80% power and α =.05.

We invited participants who completed Study 3A, 1 year after the completion of their first session. Of the original 100 participants, 52 agreed to participate. However, the addition of the Time Judgment task resulted in a high volume of technical problems, preventing 14 of these participants from completing the study.

**Participants.** The final sample of participants included 38 participants (17 women, 21 men, *M*_age_ = 36.9, *SD*_age_ = 6.0) who were paid £3. One participant only completed the DI task. All participants had normal or corrected-to-normal vision and were fluent in English.

**Apparatus.** The apparatus details were the same as in Study 3A.

#### Distractor intrusion task

***Procedure.*** Participants always completed the distractor intrusion task first and the Time Judgment task second. The distractor intrusion task was the same as the one used in Study 3A, except that each participant completed 50 trials.

***Stimuli.*** The stimuli for the distractor intrusion task were the same as Study 3A.

***Data curation.*** Practice trials were not analyzed. No participant had a score more extreme than 3 SD above or below the mean DI rates, and therefore no participants were treated as outliers.

#### Time judgment task

**Fig. 8:**
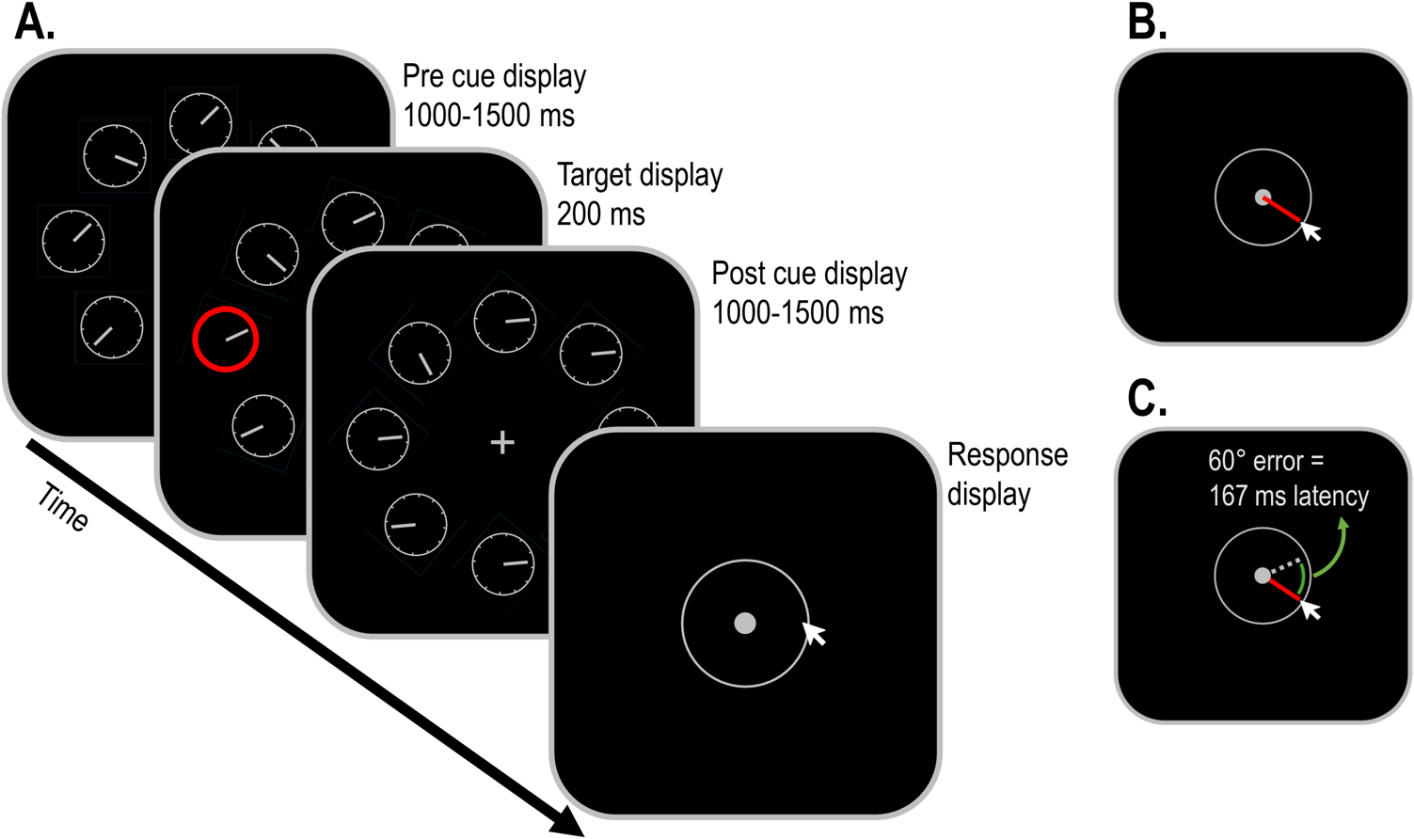
Time Judgment task. Participants viewed eight moving clocks in search of a colored cue (**A**). In the response display (**B**), they report the orientation (time) of the cued clock’s clock hand at the time of the cue’s appearance. The difference in degrees between the actual time and the reported time (**C**) reflects the time error. Any response equal to or more extreme than ± 160 degrees was discarded

***Procedure.*** For the Time Judgment task, participants were asked to observe eight clocks with rotating clock hands and to identify the time on one of the clocks when it was cued by a red circle outline. Each trial contained four displays (Fig. 8A). First, in the pre-cue display, the eight clocks rotated in tandem for a random duration of 1000–1500 ms. The starting position of each clock hand was selected at random. All the clock hands moved clockwise at a rate of one rotation per second. Second, in the cue display, a circle outline appeared around one of the clocks for 200 ms. During this time, the clock hand continued moving. Which clock was the target clock was randomly selected for each trial. Third, in the post-cue display, the cue disappeared, and the clock hand continued moving for an additional random duration of 1000–1500 ms. Finally, in the response display, participants were presented with a single empty clock and used the mouse to indicate the target clock’s time when the cue appeared (Fig. 8B). The Time Judgment task was presented in two blocks of 50 trials each, which were preceded by a block of 20 practice trials. The first practice trial had a longer (1000 ms) cue display.

***Stimuli.*** Each clock subtended 1.1° in diameter and appeared at eight equidistant positions on an invisible 4°-radius circle around fixation. The clock hands were 0.45° in length. Throughout the trial, the clock hand changed its position every frame, depending on the participants’ refresh rate to produce a speed of 1 rotation per second. E-prime go collects data about presentation refresh rates. Since all participants had 60-Hz monitors, every 16.67 ms the clock hand moved by 6°.

***Data curation and analysis.*** Practice trials were not analyzed. Analysis of the Time Judgment task focused on the difference between the time indicated by the participant (t_p_) and the time of the target clock during the cue display (t_c_). Figure 8C illustrates an example where t_c_ was set at 80 degrees (2:40 on the clockface), and t_p_ is 140 degrees (4:20 on the clockface), resulting in a time error of 60 degrees (140–80 degrees). Since a single revolution of the clock hand took 1 s, this would translate into a 167-ms delay in reporting the target. Similarly, responses indicating a time that preceded t_c_ resulted in a negative time error. Because time errors of 180° could reflect either a latency of 500 ms or −500 ms, any errors of ±160° were discarded from analysis (1.9% of trials). Next, trials were considered outliers and were excluded from analysis if they reflected an error greater than 3 SDs away from a participant’s mean time error (0.9% of remaining trials). No participant’s score was more than 3 SD above or below the sample’s mean time error, and therefore no participants were treated as outliers.

#### Results

**Fig. 9:**
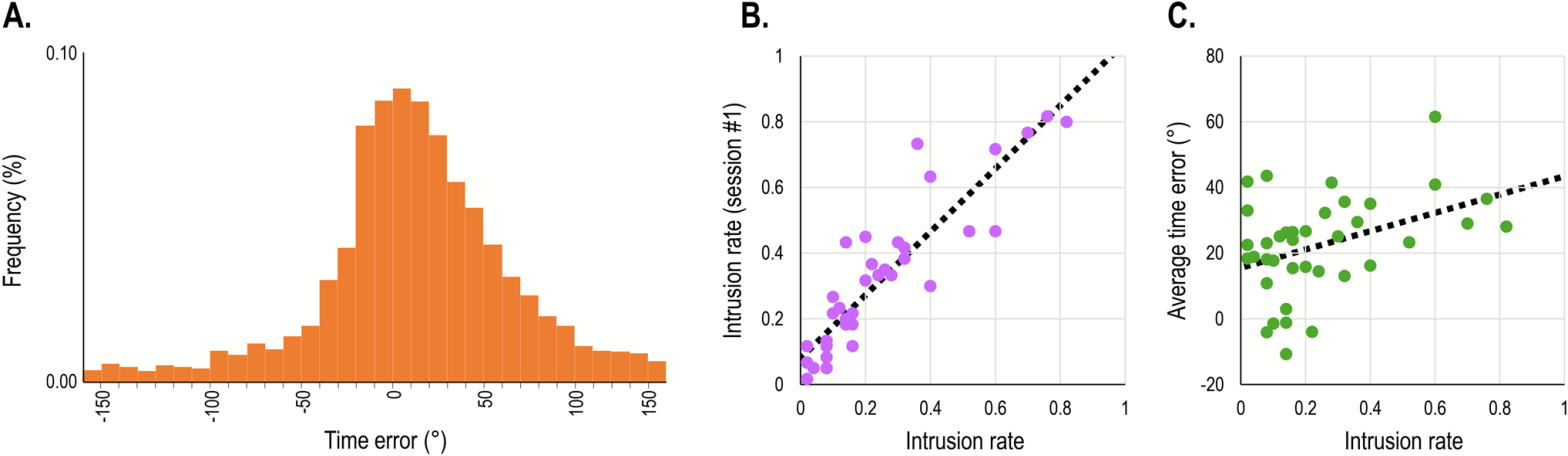
Results from Study 3B. The leftmost panel (**A**) shows the distribution of time errors in the Time Judgment task. Each bin reflects 10°. The two rightmost panels show scatterplots describing the relationship between DI rates (*x*-axis) and: (**B**) DI rates from a previous session, and (**C**) average time error in the Time Judgment task. The *dotted line* reflects the linear regression equation calculated based on these results

**Time judgment task.** The distribution of errors in the Time Judgment task is presented in Fig. 9A. The distribution was positively skewed with a mean of *M* = 22.41° (SD = 15.14), corresponding to an error of approximately 62.25 ms. The mean time error was significantly different from 0, *t*(37) = 9.12, *p* <.001, *BF*_*10*_ > 100.

**Within-session reliability.** We calculated the Spearman–Brown split-half reliability for the two main measures: time errors and intrusion rates. Within-session reliability was high for time errors, *r’* =.83 and higher still for distractor intrusions, *r’* =.96.

**Between-session reliability.** We calculated the between-session reliability for DI rate as the correlation between the DI rate in the current session and DI rate in the first session (Study 3A). Similar to the previous studies, the correlation was strong and significant, *r*(36) =.90, *p* <.001, *BF*_*10*_ > 100 (Fig. 9B).

**Correlation.** Finally, we calculated the correlation between the average time error and DI rate. The correlation was significant, *r*(36) =.40, *p* =.007, *BF*_*10*_ = 7.51 (Fig. 9C). As in Study 3A, the correlation remained significant when participants’ age was controlled for, *r*_*partial*_ (36) =.39, *p* =.008.

#### Discussion

Study 3 produced three key findings. First, the results confirmed the conclusions from Studies 1 and 2 that the DI task produces highly reliable results (*r*s ≥.90) within a single session (internal consistency), between two sessions (test–retest reliability), a week apart, and even 1 year apart. This contrasts with the attention control measures used in Study 3A, the Simon effect and the cueing effect, which (in line with previous studies, Hedge et al., 2018b; Roque et al., 2016) had lower reliability scores (*r*’ =.58 to.66).

The second key finding is that DI rates were positively correlated with tasks that are associated with the speed of attention. Higher DI rates were associated with slower RTs in the Visual Search and the Simon task (Study 3A) and with larger time errors on a Time Judgment task (Study 3B). In both cases, this relationship was independent of participants’ age. The large sample size in Study 3A allows for confidently concluding that the association between DI rates and overall RTs is weak. This is unsurprising given that overall RTs do not only reflect the speed of attention but rather the outcome of multiple processes (Palmer et al., 2011), which are not necessarily correlated with one another. A strong correlation would have been more likely to emerge if DIs rates relied on all of the same set of processes that underlie overall RT, not just the speed of attention. In Study 3B, the high attrition rate bars any meaningful conclusion regarding the absolute magnitude of the correlation between DI rates and time errors. Nevertheless, the observed correlation (supported by a Bayesian analysis) suggests that these two measures are positively associated with one another: higher DI rates are associated with larger time errors. Altogether, the results of Study 3 support the conclusion that DI rate is a valid measure of the speed of attention.

The third key finding was that DI rates were not significantly correlated with two measures of attentional control, the Simon effect and cueing effects. The absence of any significant correlation may be attributed to the difference score method required to calculate these measures and the resulting low reliability, which introduces noise and limits the likelihood of observing a correlation. However, these null results are also compatible with the view that variability in DI rates is not predominantly attributable to attentional control. One reason for this is that the DI task used here likely results in ceiling levels of attentional control, thereby minimizing related variability between participants. Indeed, it is possible that a stable correlation with attentional control will emerge in a DI task where goal maintenance and management is more challenging. In such a task, attentional control may indeed have observable effects on how quickly attentional engagement is deployed.

## General discussion

Individual differences in the speed of attention may explain real-world behavior, such as detection of road hazards (Barragan & Lee, 2021), and may predict psychological variables, such as fluid intelligence (Mashburn et al., 2024). However, research into this topic is beset by methodological challenges. Specifically, popular measures of the speed of attention rely on reaction times (RTs), and therefore suffer from issues related to either interpretability or reliability (Draheim et al., 2019; Hedge et al., 2018a; Palmer et al., 2011). The current study examined an alternative measure to RTs that is not vulnerable to these issues.

Previous experimental studies showed that Distractor Intrusions (DI), the erroneous report of a distractor in an RSVP instead of the target, measure the speed of attention (Chun, 1997; Ludowici & Holcombe, 2021; Vul et al., 2008; Zivony & Eimer, 2021; 2023). In the current study, we demonstrated that DI rates also consistently vary between participants, providing a reliable measure of individual differences in the speed of attention.

Our first goal was to demonstrate the reliability of DI rates. In three studies, DI rates were found to be a highly reliable measure, both within a single session, between two sessions a week apart, and even between two sessions a year apart. Reliability was remarkably high even for a small number of trials (50–60). This suggests that DI rates can be measured reliably with a single 5-min session. For example, in Studies 3A and 3B, completing 50 DI trials took an average of 3 min (not including instructions and five practice trials). A plausible reason for this benefit is that DI rates (unlike RTs) do not require the subtraction of raw scores, a method known to limit reliability (Caruso, 2004; Cronbach & Furby, 1970). Moreover, DI rates do not rely on speeded responses, and are therefore unaffected by speed–accuracy trade-offs and by variability in overall response speed (Draheim et al., 2019). This suggests that DI rates are a highly useful tool for individual differences research.

The second purpose of the current study was to further characterize the process reflected by DI rates, by examining the correlation between DI rates and other measures. Even though responses in the DI task are not given with time pressure, DI rates correlated with overall RTs in two attention tasks, Visual Search and Simon. DI rates also correlated with T1 accuracy in an attentional blink (AB) task and with errors on a Time Judgment task. These results are expected from a measure that indexes the speed of attention. In contrast, DI rates did not significantly correlate with reading efficiency and measures of attentional control. We also observed that DI rates are uncorrelated with individuals’ attentional blink (AB) rates, and this null finding was robustly supported by a Bayesian analysis. The AB reflects a decrement in reporting the second of two targets for a short period of time (200–500 ms) following attentional engagement and encoding of a first target. The results suggest that the two phenomena reflect separate limitations to temporal selectivity and that the two measures are not interchangeable. Whilst individual differences in AB rates are thought to reflect an individuals’ speed of encoding (Martens et al., 2006), individual differences in DI rates are more closely linked to an individuals’ speed of attention. Thus, one cannot assume that factors that affect individual differences in sensitivity to the AB (Willems & Martens, 2016) will also affect DI rates; the door is open for further research into DIs and their correlates.

One goal of such future research would be to further characterize the process reflected by DI rates. If DI rates assess the speed of attention, they should correspond to other measures related to the speed of attention, as long as they do not rely on problematic difference scores calculation (e.g., search slopes). For example, DI rates are predicted to correlate with an individual’s latency of the N2pc: participants with lower DI rates should demonstrate earlier N2pcs, indicative of a tendency to engage attention quickly (Drisdelle et al., 2016). Another avenue for future research would be to use the DI task to elucidate the processes involved in other measures of attention. For example, the d2 test of attention is a reliable test of sustained attention commonly used in various sectors, including clinical settings (Brickenkamp & Zilmer, 1998; Steinborn et al., 2018). In this test, participants go over rows of letters and discard specific targets embedded among distractors. Despite its widespread usage, some questions remain about the processes assessed by this test (da Silva-Sauer et al., 2022). For example, the d2 test relies on speeded responses and therefore it is possible that the d2 test gauges individuals’ speed of attention, not just their sustained attention. Future studies can utilize the DI task to examine this option: no correlation between d2 test measures and DI rates would indicate that the d2 test distinctly assesses sustained attention, whereas a correlation with DI rates would suggest an involvement of the speed of attention. Finally, the DI task can hopefully be helpful in future research aiming to determine what real-world behaviors and psychological variables are predicted by individuals’ speed of attention.

### Limitations and alternative accounts

The results of the current study support the view that DI rates reflect individual differences in attentional processing, and specifically, in the speed of attention. Thus, the current study dovetails with previous experimental studies that demonstrated this association (e.g., Ludowici & Holcombe, 2021; Vul et al., 2008; Zivony & Eimer, 2020; 2021; 2023). However, one limitation and one alternative account of our findings deserve discussion.

One limitation of the current work is that Studies 2A, 3A, and 3B were conducted online. While the software used in the current study measured monitor size and refresh rates, situational factors such as viewing distance, lighting conditions, or background noise could not be controlled for. For example, if lighting affects both DI rates and RTs, then two participants who completed the studies under differing lighting conditions would show a correlation between the two measures regardless of real individual differences. However, this issue cannot explain the high between-session reliability observed for DI rates; in fact, it seems that DI rates are quite robust to incidental situational factors. Thus, while we are confident about the direction and the existence of some correlations we found (e.g., between DI rate and RTs), we caution against overinterpreting their effect sizes. These effects may be smaller or larger when measured under controlled lab conditions.

**Fig. 10:**
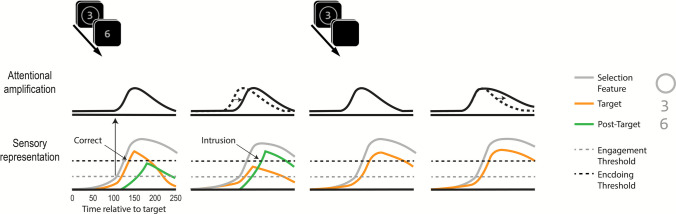
Illustration of factors that determine encoding in the diachronic account on hypothetical trials. In this example, the selection feature is a circle and the target is “3”. On the two leftmost panels (**A** and **B**) the post-target distractor is “6”, whereas on the two rightmost panels (**C** and **D**), there is no post-target distractor. The *x*-axis in each panel represents time in milliseconds from the moment signals from the target reach the visual cortex. Evidence about each feature (selection feature and identity) is accumulated separately and continuously modulated by spatially specific attentional enhancement. In addition, sensory representations mutually inhibit one another. Once the target is detected, it triggers an attentional episode. When this attentional episode is triggered early (**A**), it is more likely that the target’s features will be sufficiently strong to cross the encoding threshold and be encoded. When the attentional episode is substantially delayed (**B**), there is a higher likelihood that the post-target’s features will be encoded instead. If the target is not followed by a distractor, the sensory signal remains highly activated for a longer duration (**C**), and a delay in the offset of the attentional episode (**D**) should not affect how quickly the target is encoded

In addition, there is an alternative account of our results that is entirely compatible with our own conceptualization of attention. According to “diachronic” accounts of attention (e.g., Reeves & Sperling, 1986; Wyble et al., 2011; Zivony & Eimer, 2022), once triggered, attentional engagement enhances processing for a short period of time—an “attentional episode”. As suggested above, differences in the speed of attentional engagement can bias the perceptual competition between the target and the distractor influence and determine which object will be encoded (see Fig. 10A versus 10B). However, a delay in the *offset* of the attentional episode (rather the onset) can also increase the likelihood of DIs, as it would result in the post-target distractor benefiting from more amplification. Could differences in the offset of attention, rather than the onset of attention, explain individual differences in DI rates? We suggest that the results of Study 3A are inconsistent with this explanation. Specifically, while a later offset of the attentional episode should increase DI rates, it should not result in slower overall RTs. A delay in the offset of the attentional episode should result in poorer performance only in tasks where the target is quickly replaced by a masking object (see Fig. 10C versus 10D). This amplified distractor object then disrupts the processing and encoding of the original target. In the absence of a masking object (such as in the Visual Search and Simon tasks), delay in the episode’s offset should not result in poorer performance (and longer RTs), but rather in additional amplification of the correct target. Thus, if offset variability alone predicted individual DI rates, one would not expect DI rates to be positively correlated with overall RT in the Visual Search and Simon tasks.

Taken together, we conclude that similar to DIs in experimental studies, individual differences in DI rates measure the speed of attention. Note that this conclusion does not deny the possibility that other factors can affect DI rates or individual differences in the speed of attention under certain circumstances. Moreover, this conclusion neither challenges nor supports the diachronic account of selective attention over any other theoretical account. Instead, we only suggest that the DI task provides a robust index of the speed of attention that can be easily employed in future studies.

### Considerations using the distractor intrusion task

The DI task lends itself to a wide variety of research questions about factors that affect the speed of attention (e.g., aging, developmental conditions, sleep deprivation), and performance or psychological traits that are predicted by individual differences in the speed of attention (e.g., driving performance, fluid intelligence). To facilitate such research, we provide in the following link (https://doi.org/10.6084/m9.figshare.28376198), an easily executable experiment file for the DI task used in Study 3, alongside documentation on how to use the experiment and read the output. The experiment requires no installation and produces a.txt file that can be read by most spreadsheet software (e.g., Microsoft Excel). However, when using this experiment or when creating a new experiment where DIs is a key measure, some considerations should be kept in mind. The following is a non-exhaustive list of such considerations.

#### Between-group comparisons

**Fig. 11:**
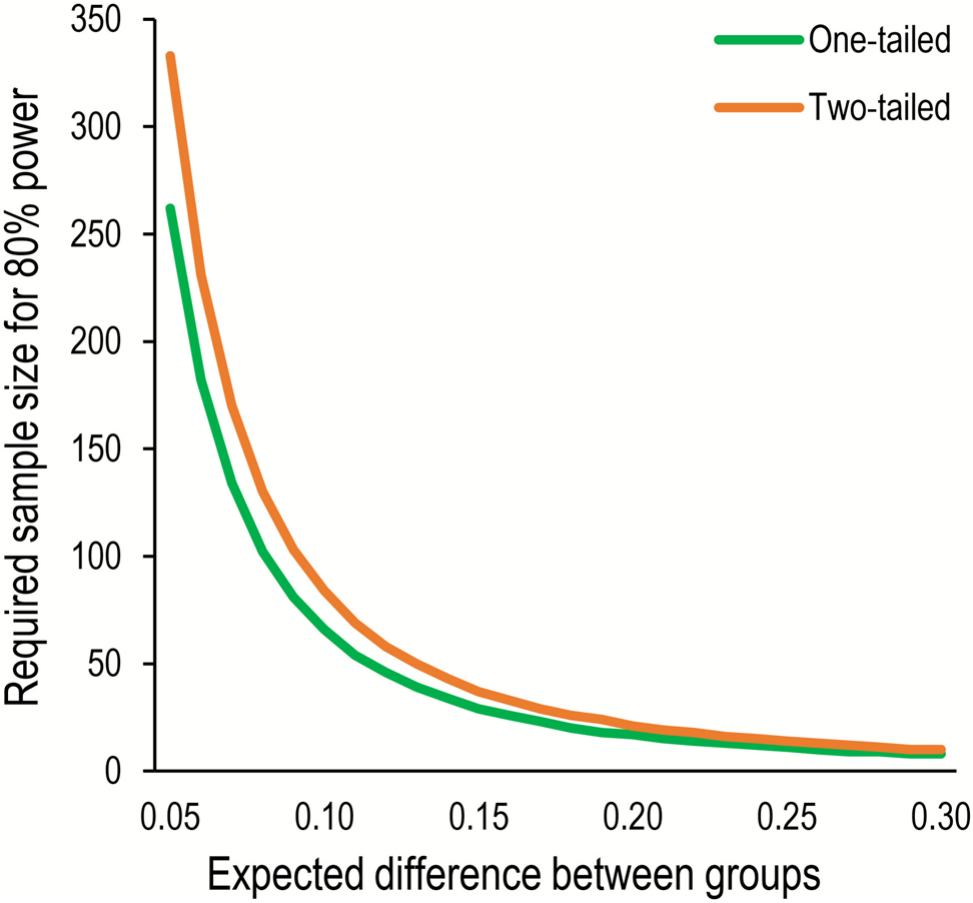
Required sample size (per-group) to achieve 80% power in a between-group comparison as a function of the expected difference between groups and hypothesis directionality (one versus two-tailed hypothesis)

DIs can be used to test for group differences in speed of attention. However, whereas the high between-subject variability in DI rates means that this measure may be suitable for individual-differences research, it also poses a challenge for studies that aim to compare groups. In a between-group comparison (e.g., using an independent sample t-test), high between-subject variability reduces the effect size. Therefore, more participants per group are needed to achieve adequate levels of statistical power. Using the data from Study 3A, we estimated that the standard deviation in intrusion rates using our experiment is σ =.23. A power analysis (conducted using the Stats package in RStudio) suggests that any between-group difference smaller than Δ =.10 would be difficult to detect, as it would require a sample size of over *n* = 100 per group (see Fig. 11). However, this estimate might be overly conservative, as a true between-groups difference may also correspond with lower within-group variability.

#### Platform

By their nature, performance in RSVP experiments is sensitive to timing (Lawrence, 1971): accuracy will be higher if the target is presented for longer durations and DI rates will be higher if the post-target distractor is presented for longer durations. We therefore recommended programming DI experiments using software or a platform that allows for a high degree of control over presentation rates. The experiments used in this study (as well as the provided example experiment) have all been programmed using E-Prime 3.0 and downloaded to participants’ computers using the E-Prime Go cloud service. Thus, even though participants completed the task on their own machines, presentation rates were independent of internet speed. Moreover, by tracking refresh rates, we were able in previous studies (e.g., Zivony & Eimer, 2024a,b) to remove the data from participants whose machines could not produce the required presentation rate. In contrast, online browser-based experiment platforms often have higher variability in presentation rates and do not necessarily track presentation times accurately. It is currently unknown to what degree random trial-by-trial variability in presentation rates is prohibitive to the usage of DI rates in individual differences research.

#### Design

Some features are known to affect DI rates, such as the presentation rate (frame duration and ISI), type of stimulus being employed, and the saliency of the selection cue (Botella et al., 2001; Vul et al., 2009; Zivony & Eimer, 2021; 2024a, 2024b). Features that are known to affect visual search, such as luminance and stimulus size (Proulx & Egeth, 2008), are also likely to play a role in DI rates. However, seemingly innocuous features of a DI task may have unforeseen consequences. In the present task, we ensured that the RSVP of distractors included potentially reportable distractors (digits) except for the distractor that immediately preceded the target, which was always non-reportable (a letter). The inclusion of reportable distractors ensured that searching for digits would be a highly unproductive strategy. Therefore, participants can only complete the task if they search for the selection cue (the circle), allowing for better experimental control over participants’ search strategy. Since participants mostly report distractors that are temporally adjacent to the target, it is highly unlikely they will report these early distractors. In contrast, the pre-target distractor in our experiments was always non-reportable to avoid pre-target intrusions. This feature was included because pre-target and post-target intrusions may actually be caused by different mechanisms (Zivony & Eimer, 2023). Therefore, the exclusion of a reportable pre-target distractor and reliance on post-target intrusion rates should produce more reliable results (Zivony & Eimer, 2024a).

#### Limitation in scope

The studies described here are all based on samples of healthy adults, whose performance in a standard RSVP task is expected to be high (see Fig. 1B). However, it’s reasonable to assume that some participants or some populations will be unable to even detect the target in the RSVP, let alone report its identity. Therefore, we recommend that future studies should exclude participants whose guess rates (i.e., reports of neither the target nor the post-target) are very high (e.g., over 25%), as their low intrusion rates may be uninformative of their speed of attention.

##### Conclusion

In the current study, we provided evidence that measuring distractor intrusions with a short (~ 5 min) task produces a highly reliable measure of the speed of attention. This suggests that the distractor intrusions task is a highly useful tool to study individual differences in attention research. We provide an example experiment that can be easily used on PCs. We hope that this will inspire researchers to use distractor intrusions in future research aimed at developing attention theories and at examining the role of attention in everyday life.

Beyond a contribution to future individual differences research, the current study can also potentially contribute to the development of theories of attention and perception. Theories of attention usually emphasize the universality of attention mechanisms. By doing so, these theories implicitly treat individual differences as random error to be averaged out. The current results show that far from random error, individual differences in the speed of attention are a major factor that determines the content of perception, deserving of serious theoretical consideration.

## Data Availability

All the data and materials necessary to reproduce the studies and analyses are available online at: 10.6084/m9.figshare.28376078

## Code availability

A distractor intrusion experiment is available at: 10.6084/m9.figshare.28376198

## Declarations

### Open data statement

All the data and materials necessary to reproduce the studies and analyses are available online at: 10.6084/m9.figshare.28376078. A distractor intrusion experiment is available at: 10.6084/m9.figshare.28376198.
